# Adsorption of Polyolefins from Alkanes on Alumina and Silica

**DOI:** 10.1021/acs.langmuir.6c03006

**Published:** 2026-10-05

**Authors:** Fawaz Motolani, Mason D. Jones, Dayrl Briggs, Bert D. Chandler, Brian K. Long, Candice Halbert, S. Michael Kilbey, Bryan D. Vogt

**Affiliations:** † Robert V. Waltemeyer Department of Chemical Engineering, 8082 The Pennsylvania State University, University Park, Pennsylvania 16802, United States; ‡ Department of Chemistry, University of Tennessee, Knoxville, Tennessee 36996, United States; § Neutron Scattering Division, Oak Ridge National Laboratory, Oak Ridge, Tennessee 37831, United States; ∥ Department of Chemistry, The Pennsylvania State University, University Park, Pennsylvania 16802, United States; ⊥ Center for Nanophase Material Science, Oak Ridge National Laboratory, Oak Ridge, Tennessee 37830, United States

## Abstract

Heterogeneous catalytic deconstruction of polyolefins offers a promising route to upcycle plastic waste into higher-value products, yet the fundamental insights needed to control product distributions remain limited. As the reactions proceed, deconstruction generates a distribution of polymer fragments and small molecules whose adsorption to catalyst supports influence accessibility to active sites and subsequent reaction pathways. Here, we use *in situ* neutron reflectivity to quantify the adsorption of polyethylene (PE) and isotactic polypropylene (iPP) onto planar alumina and silica surfaces, which serve as idealized model support surfaces for catalytic upcycling, from oligomeric alkane (dodecane and tetradecane) solutions. Fits of the reflectivity data reveal that the underlying oxide has a surprisingly large impact on the structure of the adsorbed polymer layer and the total amount of polymer adsorbed. PE adsorption from tetradecane on alumina yields relatively thin (∼2 nm) adsorbed layers that contain ∼20–40% solvent. A much thicker adsorbed PE layer from dodecane solution is observed on silica. The adsorbed PE extends tens of nanometers from the silica surface. The PE densifies near the silica surface with exclusion of alkane solvent, but there is also a diffuse layer containing >50% solvent that extends further into the solution. This structuring on the silica surface is attributed to the combination of train and loop morphologies for the adsorbed PE. For both surfaces, the total amount of PE adsorbed is greater than that for iPP under comparable conditions. Surprisingly, PE adsorption on alumina is only weakly dependent on its molar mass (10–200 kg/mol) and almost insensitive to temperature (140 vs 160 °C). These results offer fundamental insight into adsorption of polyolefins onto surfaces, which may prove useful for improving models to describe catalytic upcycling of polyolefin wastes.

## Graphical abstract

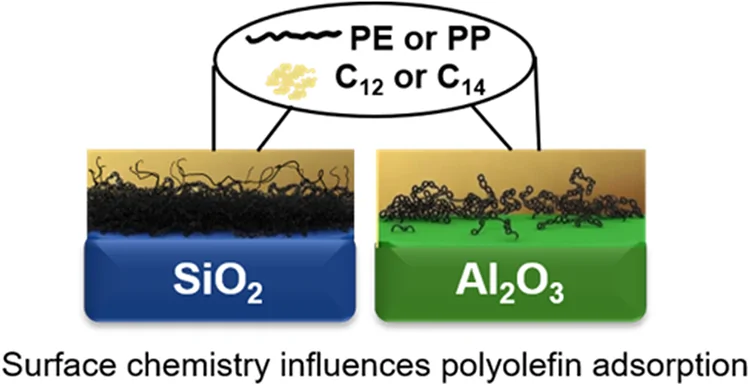

## Introduction

The rapid growth in global plastic production has created a critical waste management challenge, as most plastics are used in nondurable applications, like packaging, and disposed after a single use.^1^ These wastes are predominantly landfilled, incinerated, or leaked into the environment,^2^ contributing to pervasive pollution.^3^ Mechanical recycling, while effective for clean and well-sorted waste streams, is limited by consumer behavior in recycling, polymer degradation, contamination, and performance loss upon repeated reprocessing—these characteristics ultimately lead to downcycling rather than true circularity.^4–6^ These limitations surrounding mechanical recycling have motivated chemical recycling approaches that convert plastic waste into fuels, chemicals, or new polymer feedstocks.^7,8^ Chemical recycling can valorize mixed or contaminated waste streams through the conversion of plastic waste to smaller molecules.^9,10^ However, realizing the full potential of chemical recycling requires efficient bond scission and precise control over reaction pathways to achieve economically viable and selective product distributions.^7,8,11^

Polyolefins, primarily polyethylene (PE) and polypropylene (PP), account for more than half of global plastic production.^4^ Their chemically inert, saturated carbon backbones combined with high ceiling temperatures relative to onset temperatures for side reactions inhibit selective direct depolymerization to monomers.^7^ Thus, upcycling pathways typically convert polyolefins to alternative products, such as fuels, waxes, and specialty chemicals.^10,12^ Thermal pyrolysis has been widely explored with demonstrations at pilot and industrial scales, but is limited by poor product selectivity, high energy demands, and low material utilization, with significant production of low-value light hydrocarbons and char.^13,14^ In contrast, polyolefin deconstruction over heterogeneous catalysts enables improved product distribution control and lower operating temperatures, overcoming some of the shortcomings of pyrolysis.^15–17^ Catalytic transformations increase the yield of desired products,^15^ but these reactions are often limited by mass transfer.^18,19^ However, as the reactions proceed to deconstruct the polyolefins, smaller molecules are produced, effectively producing a polyolefin solution where polymers become solvated by the polyolefin fragments. Under these conditions, effective control of polymer adsorption and desorption from catalyst surfaces and catalyst selectivity based on molecular size become critical to avoid the production of low-value, light gases.

Computational studies have provided molecular-level insights into processes involved in the catalytic upcycling of plastics. Vlachos and co-workers demonstrated that polymer–surface interactions play a central role in chain accessibility to active sites during polyolefin deconstruction, with adsorption equilibria and chain conformations strongly influencing catalytic turnover and selectivity.^20–22^ de Gennes demonstrated that the adsorption of polymers from solution was defined by the balance of enthalpically favorable surface interactions and entropic penalties associated with chain confinement.^23^ Incorporating solvent quality, molar mass, and surface affinity into models enabled predictions of adsorbed layer thickness, segment density profiles, and adsorption reversibility for polymer adsorption from a variety of solution conditions.^24,25^ However, there are few experimental measurements of polyolefin adsorption under solvated, high-temperature conditions appropriate for understanding plastic upcycling; reported studies have focused on polar or functionalized polymers in good solvents.^26,27^ For polyolefin upcycling, the “solvent” produced *in operando* is a mixture of long alkanes, which should lead to near-athermal conditions for adsorption. Due to the large intrinsic size of polymers, polyolefin adsorption and desorption can potentially control access to active sites and ultimately product selectivity for catalytic upcycling.

Here, we use neutron reflectivity to examine the adsorption of polyolefins (PE and isotactic polypropylene, iPP) from model alkanes (tetradecane and dodecane) onto model planar oxide surfaces (silica and alumina). These surface chemistries are present on many catalyst supports, while tetradecane and dodecane serve as simple model compounds for liquid reaction products. Isotopic substitution (deuterium, D, for hydrogen, H) provides contrast in specular neutron reflectivity, enabling depth-dependent compositions of films and buried interfaces to be determined. Here, the use of deuterated alkane solvents and protonated polyolefins enables quantification of the average polymer volume fraction as a function of distance from the surface. The influence of temperature, PE molar mass, and polymer identity (iPP vs PE) on the adsorption on both silica and alumina is quantified. Based on the small change in the thermodynamics of PE solutions with decane and hexadecane^28^ and the similarities between long alkanes adsorbed on zeolite Y in the liquid state,^29^ we hypothesize that the differences in the adsorption behavior of the polyolefins will be impacted marginally by the selection of dodecane or tetradecane as the solvent. The oxide surface chemistry appears to strongly influence adsorption, with silica promoting substantially greater interfacial polymer enrichment than alumina under similar conditions. Increasing the molar mass of PE increases the mass of adsorbed polymer under the same conditions, but there is little change in the total polymer adsorption as temperature increases from 140 to 160 °C. These measurements provide new insights into the role of surface chemistry in polymer–surface interactions within nonpolar, nearly athermal environments. Although real catalyst systems are commonly particles with multiscale roughness, porosity, and active catalyst sites that alter interactions between the polymer and the support surface, these model neutron reflectivity experiments provide some insights into potential differences in surface accessibility from polyolefin adsorption simply through changing the support, which may provide a route to impact selectivity in catalytic chemical recycling.

## Experimental Section

### Materials

Single-side-polished silicon wafers (50.8 mm diameter, 5 mm thickness) were purchased from EL-CAT Inc. and used as substrates for oxide deposition. Fully deuterated *n*-tetradecane (*n*-C_14_D_30_, 98.5% D, lot no. EF-208) was sourced from CDN Isotopes. Fully deuterated *n*-dodecane (*n*-C_12_D_26_, 98% D, batch no. B003688) was obtained from Cambridge Isotope Laboratories. A set of polyethylenes (*M*_*n*_ = 10, 34, and 207 kg/mol) and isotactic polypropylenes (*M*_*n*_ = 3.7, 23, and 65 kg/mol) were synthesized via the coordination polymerization of ethylene or propylene, respectively, as described below. Precatalysts *rac*-ethylenebis­(4,5,6,7-tetrahydro-1-indenyl)zirconium dichloride, *rac*-dimethylsilylbis­(1-indenyl)zirconium dichloride, and tris­(pentafluorophenyl)­borane (B­(C_6_F_5_)_3_) were purchased from Strem Chemicals, stored in a glovebox, and used as received. Pyridylamido hafnium complex (**Hf-1**) was synthesized as previously reported.^30^ Polymethylaluminoxane-improved performance (PMAO-IP) was purchased from AkzoNobel Specialty Chemicals and used as received. Diethyl zinc (ZnEt_2_) was purchased from Fisher Scientific and used as received. Toluene was purchased from VWR Chemicals, dried via passage through an Innovative Technologies PurSolv solvent purification system, and degassed prior to use.

### Synthesis of Polyethylene (PE)

All PE samples were synthesized under identical conditions, but with varying amounts of diethyl zinc (ZnEt_2_) added as a chain transfer agent. As an example, **PE1** was synthesized using the following procedure. In a nitrogen-filled glovebox, a 6 oz. flat-bottom Fischer–Porter reaction vessel was charged with toluene (120 mL), PMAO-IP (2.31 M solution in toluene) (4.3 mL, 10.0 mmol, 1000 equiv), and a magnetic stir bar. In a separate vial, a stock solution of ZnEt_2_ (0.20 mL, 2.0 mmol) in toluene (5 mL) was prepared, and a 0.19 mL (0.07 mmol, 7.3 equiv) aliquot was transferred to the reaction vessel. Also in a separate vial, *rac*-ethylenebis­(4,5,6,7-tetrahydro-1-indenyl)­zirconium dichloride (4.3 mg, 0.01 mmol) was dissolved in toluene (5 mL) and added to the reaction vessel in one portion. The reaction vessel was sealed, removed from the glovebox, placed in a water bath at 25 °C, and equilibrated for 10 min. The reactor was then exposed to an ethylene feed (15 psi) to commence polymerization. The polymerization proceeded for 15 min before the ethylene feed was stopped and the reaction was quenched by the addition of a 2 mL aliquot of a previously prepared 1 M HCl/MeOH solution. The resulting solution was precipitated into a large volume of 1 M HCl/MeOH and stirred for 2 h, and then the solids were collected via vacuum filtration. The resulting polymer was washed with excess MeOH, collected, and dried in a vacuum oven at 60 °C overnight to obtain **PE1** as a colorless powder (*M_n_* = 34.8 kg/mol and *Đ* = 1.8). **PE2** was synthesized similarly but using a 0.72 mL (0.14 mmol, 14 equiv) aliquot of the ZnEt_2_ stock solution (0.20 mL, 2.0 mmol) in toluene (10 mL), to yield **PE2** as a colorless powder (*M_n_* = 10.9 kg/mol, *Đ* = 1.8). **PE3** was synthesized in the same fashion except without the use of ZnEt_2_ to yield **PE3** as a colorless powder (*M*_*n*_ = 207 kg/mol, *Đ* = 1.6).

### Synthesis of Isotactic Polypropylene (iPP)

All syntheses were performed in a nitrogen-filled glovebox unless otherwise noted. In a 6 oz flat-bottom Fischer–Porter reaction vessel equipped with a magnetic stir bar, toluene (120 mL) was charged along with the appropriate catalyst system as described below. The sealed reactor was removed from the glovebox, equilibrated in a water bath at a specified temperature (indicated below) for 10 min, and polymerization was initiated by exposure to propylene gas (15 psi). After the designated reaction time, polymerization was quenched by addition of 2 mL of 1 M HCl in MeOH. The reaction mixture was precipitated into a large volume of 1 M HCl in MeOH, stirred for 2 h, collected by vacuum filtration, washed thoroughly with MeOH, and dried under vacuum at 60 °C overnight. The synthetic procedures and catalysts were changed to achieve different molar masses of iPP.

For the low molar mass iPP, the reaction vessel was charged with PMAO-IP (2.31 M in toluene, 4.3 mL, 10.0 mmol, 1000 equiv). A stock solution of ZnEt_2_ (0.20 mL, 2.0 mmol in 10 mL toluene) was prepared separately, and 0.31 mL (0.06 mmol, 6 equiv) was added. *rac*-Ethylenebis­(4,5,6,7-tetrahydro-1-indenyl) zirconium dichloride (4.3 mg, 0.010 mmol) dissolved in toluene (5 mL) was added in one portion. Polymerization was conducted at 25 °C for 15 min. After precipitation, washing, and drying, **iPP1** was obtained (*M*_*n*_ = 3.7 kg/mol, *Đ* = 2.1).

For the intermediate molar mass, the reaction vessel was charged with PMAO-IP (2.31 M in toluene, 4.3 mL, 10.0 mmol, 1000 equiv). *rac*-Dimethylsilylbis­(1-indenyl) zirconium dichloride (4.5 mg, 0.010 mmol) dissolved in toluene (5 mL) was added in one portion. Polymerization was conducted at 30 °C for 1 h. After precipitation, washing, and drying, **iPP2** was obtained (*M*_*n*_ = 23.2 kg/mol, *Đ* = 1.8).

For the highest molar mass, the reaction vessel was charged with B­(C_6_F_5_)_3_ (10 mg, 0.020 mmol) dissolved in toluene (5 mL), followed by addition of **Hf-1** (13 mg, 0.020 mmol) dissolved in toluene (5 mL). Polymerization was conducted at 25 °C for 15 min. After precipitation, washing, and drying, **iPP3** was obtained (*M*_*n*_ = 65.1 kg/mol, *Đ* = 1.7).

All polyethylene and polypropylene samples were analyzed by high-temperature size exclusion chromatography (HT-SEC, Malvern Viscotek 350B high-temperature SEC) using 1,2,4-trichlorobenzene (TCB) stabilized with butylated hydroxytoluene (BHT) (100 ppm) as the eluent, one PLgel Olexis (PL1110–1400) guard column and two PLgel Olexis columns (PL1110–6400; *M*_w_ lower limit: 500 g/mol; *M*_w_ upper limit: 10,000,000 g/mol). The HT-SEC columns were heated to 150 °C, and a flow rate of 1.0 mL/min was used for the measurements. The injection volume was 200 μL. Number-average molar mass (*M*_*n*_), weight-average molar mass (*M*_w_), and dispersity (*Đ* = *M*_w_/*M*_*n*_) are reported from triple detection data (Figures S1 and S2): refractive index, viscometry, and light scattering detectors (right-angle and 7° low-angle) were used, and results were analyzed with the OMNISEC software. The triple detection system was calibrated using a narrow-dispersity polystyrene (*M*_*n*_ = 96.2 kg/mol, *Đ* = 1.02) standard.

### Atomic Layer Deposition of Silica and Alumina

Silica (SiO_2_) and alumina (Al_2_O_3_) thin films were deposited on single-side polished silicon wafers (50.8 mm diameter, 5 mm thickness) using plasma-enhanced atomic layer deposition (PEALD, Kurt J. Lesker ALD150LX system) at the Center for Nanophase Materials Science (CNMS) at Oak Ridge National Laboratory (ORNL). All films were deposited at a substrate temperature of 200 °C and a chamber pressure of approximately 800 mTorr. Film growth was carried out for 110 PEALD cycles, with each cycle lasting approximately 18 s. The resulting films had thicknesses in the range of 6.5–14 nm, as measured by spectroscopic ellipsometry. SiO_2_ films were deposited using tris­(dimethylamino)­silane (3DMAS) as the silicon precursor and ionized oxygen as the oxidant. Al_2_O_3_ films were deposited using trimethylaluminum (TMA) and ionized oxygen as the oxidant.

### Neutron Reflectivity

Neutron reflectivity experiments were carried out on the Liquids Reflectometer at the Spallation Neutron Source (SNS) at ORNL. A sealed liquid cell was used for *in situ* neutron reflectivity measurements.^31^ The cell consisted of an aluminum base supporting a single-sided polished silicon wafer coated with either alumina or silica, with the polished surface forming the reflecting interface. A Kalrez O-ring was positioned on top of the coated wafer to define and seal the liquid reservoir that was created when a second silicon wafer (5 mm thickness) was placed atop the O-ring. This stack (aluminum base, wafer, fluid gap, wafer) is covered by an aluminum top plate that is equipped with two injection ports that align with through-holes in the second silicon wafer, which enables the addition of a liquid. The assembled cell was mechanically clamped to ensure a leak-tight seal and mounted on a temperature-controlled sample stage.

Polyethylene (PE) and isotactic polypropylene (iPP) were selected as representative polyolefins relevant to catalytic chemical recycling. Deuterated *n*-tetradecane (dC_14_) and *n*-dodecane (dC_12_) served as model liquid reaction products and provided neutron contrast through isotopic substitution. These two solvents were selected on the basis of their availability and normal boiling points relative to the measurement temperatures. The use of hydrogenated polymers with deuterated solvents enabled direct measurement of polymer adsorption by neutron reflectivity while maintaining chemically equivalent environments. Polymer solutions were prepared at 0.4 wt % for each polyolefin using the perdeuterated alkane solvents. Solutions of polyethylene (PE; *M_n_* = 10, 34, and 207 kg/mol) were prepared in deuterated *n*-tetradecane (dC_14_) and deuterated *n*-dodecane (dC_12_), while isotactic polypropylene (iPP; *M_n_* = 3.7, 23, and 65 kg/mol) was dissolved in deuterated *n*-dodecane (dC_12_). All solutions were prepared at 140 °C to ensure complete dissolution.

*In situ* neutron reflectivity (NR) measurements were conducted by injecting hot polymer solutions (140 °C) into the preheated cell at 140 °C to maintain polymer solubility and avoid precipitation of the polyolefins in the cell. NR measurements were performed initially at 140 °C; the sample was subsequently heated to 160 °C and a second set of NR measurements were performed. The temperatures are limited by the heating capacity of the cartridge heaters embedded in the aluminum base and limitations of the O-rings and other components in the cell. Reflectivity data were collected at incident angles of 0.45, 1.2, and 3.5° using wavelengths from 0.30 to 0.96 nm with a center wavelength of 0.62 nm at 30 Hz, which corresponds to a momentum transfer vector (*Q* = 4πsin­(θ)/λ) range of 0.1–3.0 nm^–1^. Data from the different angles were combined into a single reflectivity profile for each contrast condition and temperature. Each measurement required approximately 3 h of acquisition time, during which the sample temperature was maintained constant.

Reflectivity data were analyzed using the REFL1D software package.^32^ Prior to polymer adsorption measurements, bare oxide-coated silicon substrates were measured using either spectroscopic ellipsometry (M-2000, J.A. Woollam, Inc.) or neutron reflectivity to determine the oxide layer thickness and interfacial roughness (Figure S3). The layer was described by its thickness, scattering length density (SLD), and interfacial roughness. The SLD of the alumina layer was allowed to vary within physically reasonable constraints (4.17–4.284 × 10^–4^ nm^–2^) based on previous reports for Al_2_O_3_. These substrate parameters were then fixed for fitting the polymer adsorption. The SLD of the polymer layer was constrained by the known scattering length densities of the polymer and the solvent used. Table 1 summarizes the SLD of each solvent at temperatures used for the NR measurements, as calculated from the known elemental scattering lengths for the elements^33^ and the density of the pure component at the measurement temperature. For the polyolefins, their reported melt densities, 0.76 g cm^–3^ for PE and 0.739 g cm^–3^ for iPP, were used.^34,35^ The density change of polyolefins over 20 °C is within the uncertainty of their reported temperature-dependent density.^36^ When considering the thermal expansion of PE melts, the difference in SLD at 140 and 160 °C is less than 2%. Therefore, a constant SLD for the polyolefins was used for all fits, as listed in Table 1. The reflectivity data were fit by minimizing the goodness of fit, expressed by χ^2^, between calculated and measured reflectivity profiles; additional layers to describe the adsorbed polymer were added until a significant improvement in the fit quality was not obtained by the addition of a layer. These fits were repeated multiple times from different initial starting conditions to ensure the fits were robust, as determined from the reproducibility of the SLD profiles obtained from the fits. One example of this repetitive fitting is shown in Figure S9.

**Table 1:** Neutron Scattering Length Density (SLD) of Solvents and Polyolefins Used to Analyze the Reflectivity Data

**component**	**SLD**_**i**_ **(×10** ^**–4**^ **nm** ^**–2**^ **)**
dC_12_ (140/160 °C)	5.882/5.734
dC_14_ (140/160 °C)	5.961/5.820
PE (constant)	–0.272
iPP (constant)	–0.265

The fit of the reflectivity provides the SLD profile as a function of distance from the substrate. This SLD profile was converted to a polymer volume fraction profile assuming ideal solution mixing between polymer and solvent as

1 $$\Phi_{Ppolymer}=\frac{{SLD}_{layer}\left(z\right)-{SLD}_{solvent}}{{SLD}_{polymer}-{SLD}_{solvent}}$$

where Φ_polymer_ is the volume fraction of either PE or iPP, SLD_layer_(*z*) is the SLD from the model fit at position *z* from the substrate surface, and SLD_polymer_ and SLD_solvent_ denote the scattering length densities of the polymer (PE or iPP) and deuterated solvent, respectively, at the conditions for the measurement (see Table 1 for the SLDs used).

## Results and Discussion

The adsorption of polyethylene (PE) and polypropylene (iPP) from heavy alkanes, as shown in Figure 1a, onto silica or alumina films on silicon wafers was probed using specular neutron reflectivity. Figure 1b provides a schematic of the sample cell used for the neutron reflectivity (NR) measurements. The bottom silicon wafer with an ALD-deposited layer of SiO_2_ or Al_2_O_3_ was exposed to dilute (0.4 wt %) polyolefin solutions in deuterated alkanes at elevated temperatures. The incident neutron beam enters through the bottom silicon wafer and reflects from the solution-wafer interface. The intensity of the specular reflected beam was measured to obtain the reflectivity (*R*(*Q*) = *I*(*Q*)/*I*(0)) as a function of the momentum transfer vector, *Q*. The specular reflectivity provides information about the spatial distribution of scattering length density (SLD) that is perpendicular to the substrate surface.

**Figure 1:**
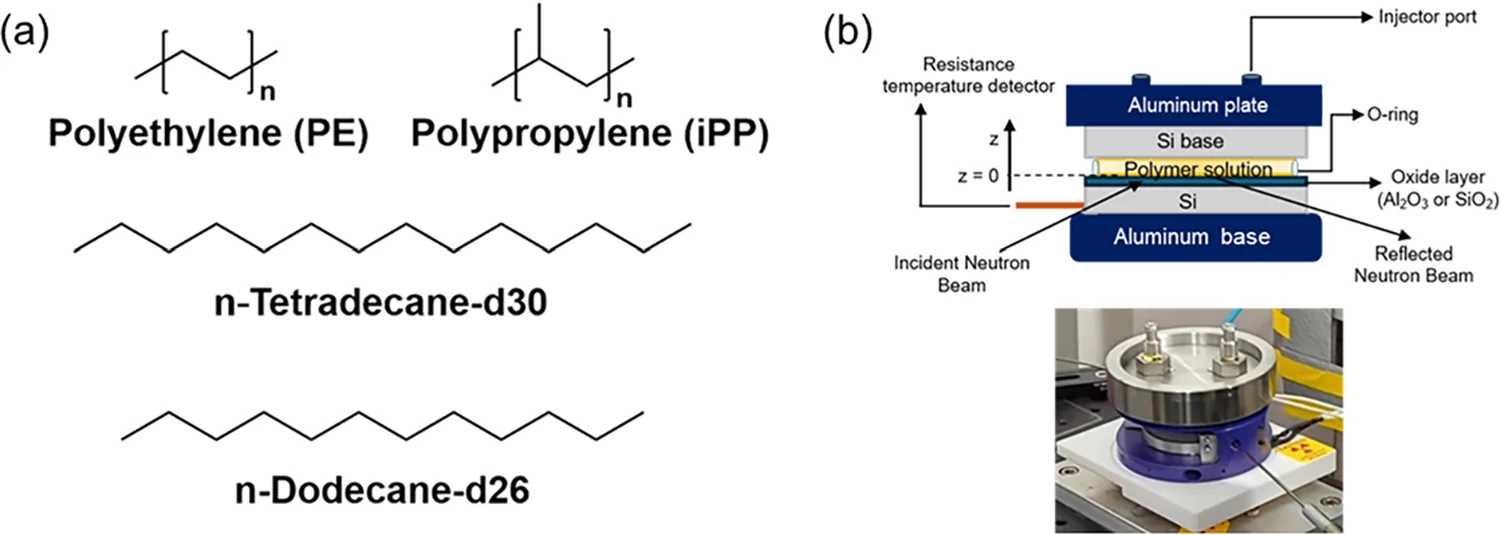
(a) Chemical structures of polyethylene (PE), isotactic polypropylene (iPP), and the deuterated alkane solvents used in this study (*n*-tetradecane-d_30_ and *n*-dodecane-d_26_). (b) Schematic of the liquid cell and reflectivity geometry used, along with a picture of the assembled cell.

### Neutron Reflectivity Profiles for PE Adsorbed on Al_2_O_3_ and SiO_2_

Figure 2a shows the measured neutron reflectivity and corresponding fits for the 34 kg/mol PE adsorbed from deuterated tetradecane on Al_2_O_3_ (adsorption conditions: 0.4 wt % PE in dC_14_, 140 and 160 °C). The data are presented as *RQ* ^4^ to better illustrate the quality of the fit in the mid- and high *Q*, where diffuse adsorbed layers tend to have the largest impact on the reflectivity fits.^37^ Models containing only the substrate, oxide, and bulk solvent fail to describe the measured reflectivity. Inclusion of multiple layers in the recursive model, which describes polymer-enriched interfacial layers, significantly improves agreement between the model and the experimental data.

**Figure 2:**
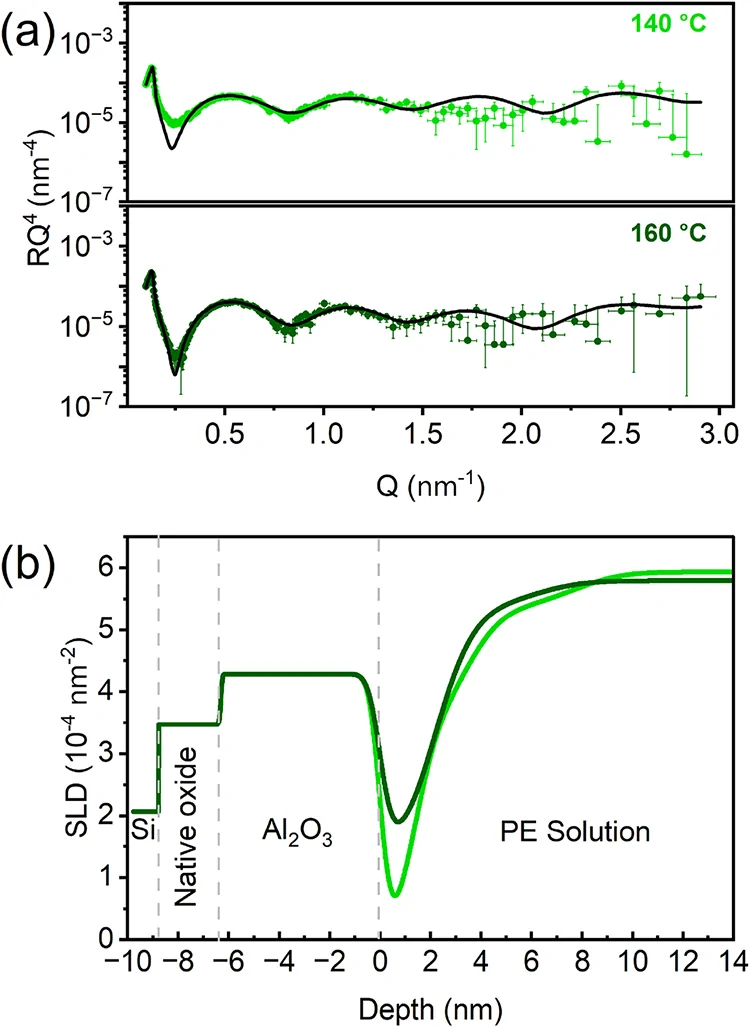
(a) Neutron reflectivity (NR) data (symbols) and model fits (solid lines) for the adsorption of 34 kg/mol PE from 0.4 wt % dC_14_ solution on Al_2_O_3_ at 140 °C (bright green) and 160 °C (dark green). (b) SLD profiles from the fits at 140 °C (bright green) and 160 °C (dark green).

The SLD profiles derived from these fits (Figure 2b) show that the SLD decreases substantially in the first 2–3 nm from the Al_2_O_3_ surface/PE solution interface. This occurs because the neutron SLD for polyethylene is lower than that for the deuterated solvent (Table 1), so the initial SLD decrease from the Al_2_O_3_ interface is attributed to enrichment of polyethylene at the surface. There is an appreciable tail in the SLD as the distance from the surface is increased, suggesting a diffuse interface between the adsorbed layer and the bulk solution. Increasing the temperature from 140 to 160 °C induces a slight shift in the Kiessig fringes in the reflectivity data and a concomitant increase in the SLD. This indicates a loss of near-surface PE, which is consistent with temperature-driven release of the adsorbed PE into the bulk solvent.

Figure 3a shows the neutron reflectivity curves and corresponding fits for the same 34 kg/mol PE adsorbed onto SiO_2_ under similar conditions (0.4 wt % PE in dC_12_, 140 and 160 °C). The data is more challenging to fit than the analogous reflectivity for Al_2_O_3_. The Kiessig fringes in the reflectivity data on SiO_2_ appear to be associated with convolution of multiple periods unlike the simple beating pattern in the reflectivity profiles from adsorption on Al_2_O_3_. These differences suggest PE adsorption on SiO_2_ is more stratified, with the adsorbed layer being less diffuse. At 140 °C, the data near *Q* ≈ 0.8 nm^–1^ could not be fit by including additional interfacial layers or redistributing the SLD among thinner layers without loss of the other features in the reflectivity. The data in the region that is challenging to fit does not contain any clear features, which suggests that the inability to fit the full data set may be associated with an evolution in the adsorbed layer structure during the measurement. The solution is dilute, but as the PE adsorbs on the surface, the local concentration increases, which can lead to slow dynamics. The structure of adsorbed polymer chains is known to evolve over 100s of hours on annealing in the melt.^38^ Heating the sample to 160 °C induces a large shift in reflectivity, with the fringes at low *Q* becoming better defined, suggesting a change in the structure of the adsorbed layer. The reflectivity data obtained on subsequent heating to 160 °C is fit much better to the model with a negative SLD due to adsorption of PE near the silica surface, as shown in Figure 3b. The minimum in SLD is nearly the same as the SLD of pure PE (Table 1). The high polymer concentration of the adsorbed layer may lead to slower dynamics in this system and explain the poor fit of the data near *Q* ≈ 0.8 nm^–1^ at 140 °C.

**Figure 3:**
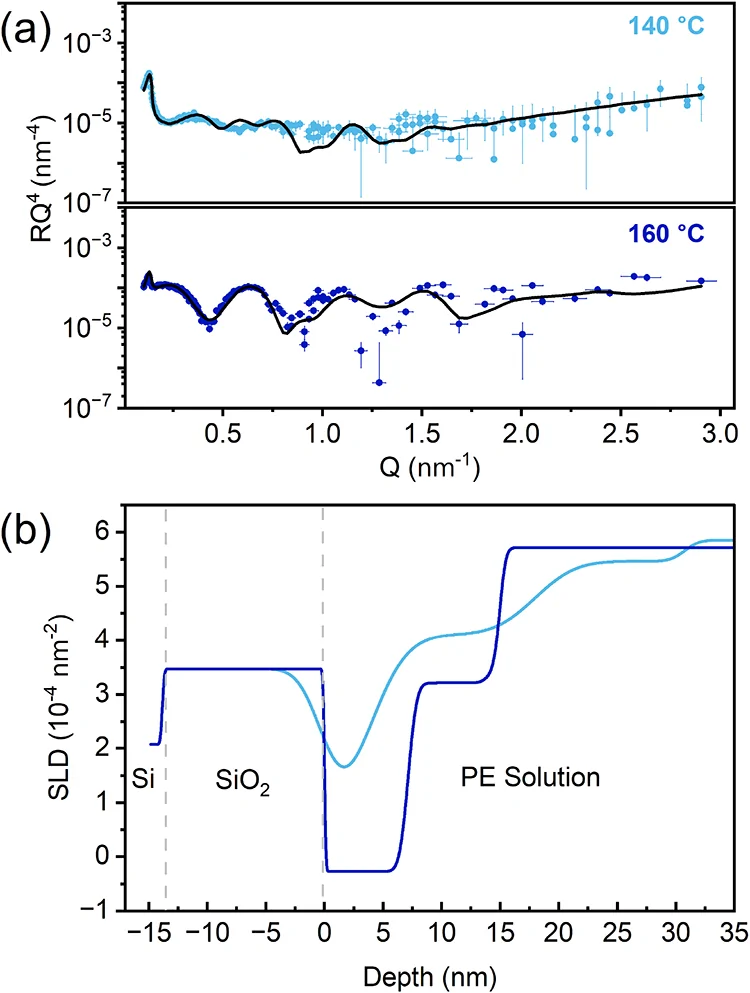
(a) Neutron reflectivity (NR) data (symbols) and model fits (solid lines) for 0.4 wt % 34 kg/mol PE in dC_12_ adsorbed on SiO_2_ at 140 °C (blue) and 160 °C (dark blue). (b) SLD profiles normal to the interface obtained from fitting the data acquired at 140 °C (blue) and 160 °C (dark blue).

Examination of the SLD profiles provides some insight into the structure of the adsorbed layer. The fit of the reflectivity data uses layers to describe the average SLD in the plane of the interface, so the layers included are not discrete physical adsorption layers, but describe the average polymer concentration as a function of distance normal to the surface. At 140 °C, a low SLD associated with a polymer-rich layer extending ∼5 nm from the silica interface is deduced, with a slightly higher SLD but still a polymer-rich layer spanning an additional ∼10 nm from the substrate as a weakly adsorbed layer. As shown in Figure 3b, the adsorbed PE appears to become more stratified on heating to 160 °C. However, the reflectivity at 140 °C is likely not at equilibrium, so the increased stratification may simply be a result of kinetic control of the adsorbed layer. The loss in the diffuse “tail” could be attributed to removal of weakly adsorbed PE, consistent with enhanced solubility at higher temperatures, while the remaining adsorbed PE is effectively tethered to the SiO_2_ to prevent chains from extending away from the substrate. However, the large contrast in the system leads to poor sensitivity in the fits to a long, diffuse, and dilute PE tail from the surface, so we cannot conclude that there is not a small concentration of adsorbed PE that extends into the bulk solution. One additional feature is the change in the width of the interface between the polymer and silica surface due to a change in SLD at the interface due to adsorption. This can effectively appear to alter the roughness of the silica surface due to the use of an error function in the recursive model to describe the interface. However, this change in the interface reflects the polymer concentration gradient described by the fit of the roughness of the layer in contact with the silica rather than a change in the physical roughness of the SiO_2_ substrate.^39,40^

### Structure and Composition of Adsorbed PE Layers

The SLD profiles were converted to volume fraction of PE using eq 1 to quantify the adsorbed PE layers (Figure 4). The differences between adsorption on silica and alumina surfaces are significant; we examine adsorption on alumina first as a baseline. At 140 °C, the PE layer near the Al_2_O_3_ surface is nearly 80 vol % PE (Figure 4a), which corresponds to a strongly adsorbed layer. Note that there will be a depletion layer when a chain is adsorbed to a hard wall due to segmental conformation restrictions. However, convolution of the interfacial roughness in the profiles from the fits produces the minimum feature in the volume fraction of PE at the substrate surface, which is sensitive to small changes in the fit of the reflectivity data that do not impact the goodness of the fit. Thus, the extent of depletion of polymer directly at the interface compared to the maximum adsorption is uncertain. However, the adsorbed polymer largely excludes solvent molecules near the interface, but the relative amount of solvent incorporation increases quickly further from the surface. This indicates the polymer adsorbs as a ∼2 nm layer that rapidly becomes more diffuse (or increasingly porous) away from the surface. There is an additional feature in both profiles, a small shoulder that corresponds to a dilute outer region.

**Figure 4:**
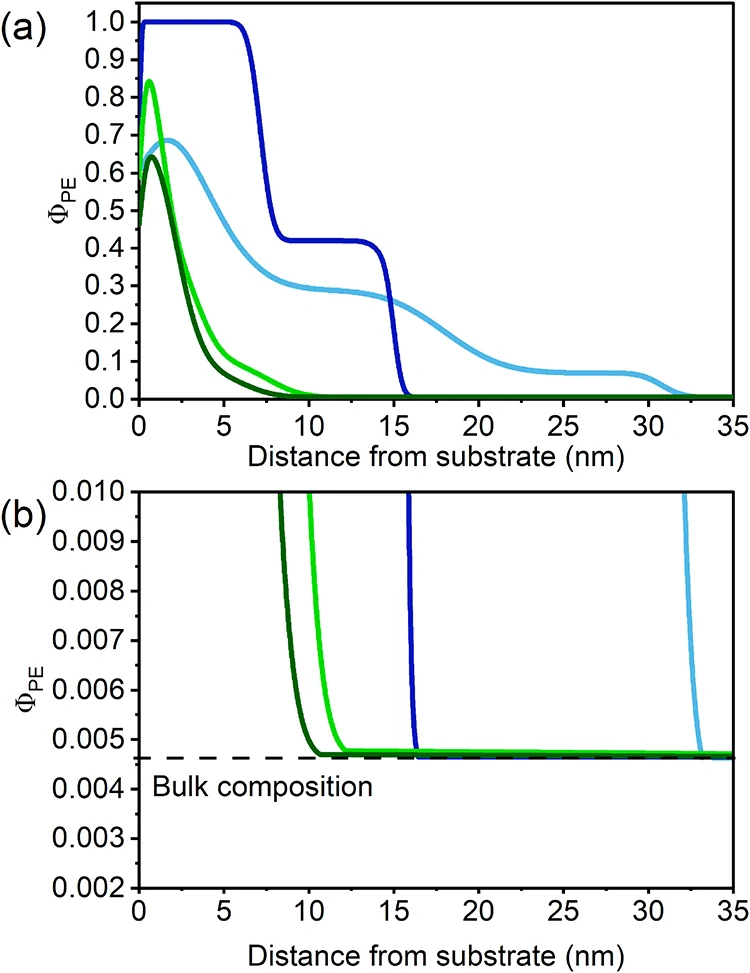
(a) Polyethylene volume fraction (Φ_PE_) profiles as a function of distance from the substrate for Al_2_O_3_ (green, 140 °C; dark green, 160 °C) and SiO_2_ (light blue, 140 °C; dark blue, 160 °C) surfaces. The near-surface maximum in Φ_PE_ arises from convolution of the SLD with interfacial roughness of the oxide surface. (b) Expanded view highlighting the decay of Φ_PE_ toward the bulk solution composition at larger distances from the interface.

Increasing the temperature to 160 °C induces relatively minor changes to the adsorbed layer. The same general structure (dense interfacial layer decaying quickly into the solvent) persists. The maximum PE concentration near the alumina surface drops by approximately 10% at the higher temperature, suggesting weaker adsorption of the polymer with more solvent incorporated in the gel-like layer at higher temperatures, but a small shoulder in the PE concentration at 5–7 nm from the interface can be seen for the PE on alumina. This feature evolved on heating from 140 to 160 °C with a decrease in the PE concentration and extension of PE chains further from the substrate. The decrease in interfacial enrichment near the surface at 160 °C can be interpreted within the train–loop framework of polymer adsorption.^41,42^ Strongly adsorbed chains contain a higher fraction of surface-contacting trains, whereas loosely adsorbed chains contain more extended loops. At elevated temperature, the balance between attractive segment–surface interactions and the entropic penalty of chain confinement shifts, due to weakening of the “sticking energy” and increase in mobility of the chains that reduces the stability and population of surface-contacting trains. As a result, the interfacial layer becomes less dense, but extends slightly more into the solution. This extension of the less dense layer can be resolved by examining the change in the shoulder of the PE concentration profile on heating from 140 to 160 °C. These results are consistent with prior studies for other polymer–solvent systems that report increased segment detachment or partial desorption with increasing temperature.^43,44^

At 140 °C, the structure of the adsorbed PE layer (34 kg/mol) on silica is generally similar to the PE layer on alumina (dense interfacial layer decaying into the bulk solvent) with two important differences. First, the adsorbed PE layer extends farther from the surface, with the SLD not matching the bulk solution until about 35 nm from the surface. Second, the adsorbed PE layer on silica has three distinct regions: (i) a near-surface region of high polymer density, which is comparable to PE/alumina system and extends ∼10 nm from the surface; (ii) a layer from 10 to 20 nm from the surface consisting of ∼30% polymer; and (iii) a diffuse layer that extends to ∼35 nm and exhibits a monotonic decay to the bulk solution composition. It is important to note the likely slow kinetics of adsorption at 140 °C for the PE on silica suggests that the structure is not the equilibrium morphology. Nonequilibrium structuring of polyolefins is likely to be present in actual chemical recycling processes that depend on the catalyst turnover rate relative to the dynamics of the adsorption. The profiles presented here appear to be at equilibrium except for the PE adsorption on silica at 140 °C, so the concentration profiles in general represent the maximum coverage of the surface for a planar surface. For real catalyst systems, the adsorption may be further altered by geometry changes with curved and rough surfaces, porosity that impacts molecular mass-dependent partitioning,^45^ and dispersed active catalysts.

The interfacial profile of PE/SiO_2_ undergoes a dramatic change upon increasing the temperature to 160 °C: The adsorbed layer forms a stratified two-layer structure. The layer closest to the silica, which is about 5 nm in thickness, appears to be nearly pure polyethylene (Figures 3b and 4a). The second layer has lower segmental density (∼40% PE) compared to the interfacial region. At the edge of the second, less dense layer, the polymer fraction rapidly decays to the bulk composition. The origin of these apparent layers is likely associated with strongly adsorbed chains nearest to the substrates, where there is a significant fraction of trains with coexistence of weakly adsorbed chains that contain predominantly loops, which can extend further from the substrate.^46^ Annealing of polystyrene on silicon wafers leads to a similar stratification as observed here.^38^ Moreover, the generation of stratified adsorbed structures is consistent with prior work from Granick and co-workers examining polymer adsorption from dilute solutions.^47–50^ The polymer near the surface would be expected to adsorb strongly as flattened chains with a high fraction of trains, while additional chains would adopt a more weakly bound loop- and tail-rich conformations that penetrate further into the solution.^47–50^ More recent simulations examining the adsorption of polymers on biological substrates^51^ found a similar structure with origins consistent with the coexistence of trains and loops at the surface.

The differences in interfacial structure seen for the two model substrates indicate that the nature of the oxide surface has a profound impact on the amount of polymer adsorbed, the structure of the adsorbed layer, and the temperature-dependent behavior of these layers. The observed effects are considerably larger than we expected for the silica surface and indicate that details of the interactions between the oxide and polyolefin can impact the system tens of nanometers from the oxide surface. The adsorption of polymers on surfaces has been shown to depend predominantly on differences between the polymer–surface interaction and solvent–surface interaction.^52^ The solvent–surface interaction appears to be modestly impacted by the change between tetradecane and dodecane,^29^ so we infer that the surface chemistry is driving the differences in the adsorption between alumina and silica surfaces.

The stronger adsorption of polyethylene on SiO_2_ relative to Al_2_O_3_ in nonpolar alkanes can be rationalized by differences in oxide surface chemistry and the nature of polymer–surface interactions. The low dielectric constant of the alkanes and PE provides a large barrier to dissociation and polarization, so the interaction between these hydrocarbons and oxide surfaces should be weak, induced dipoles. This is similar to deprotonation of zeolites depending on the dielectric constant of the fluid.^53^ However, adsorbed water on the oxide surfaces is difficult to remove, and thus residual water on the surface can act to polarize the surface. The hydroxylated SiO_2_ surface presents the polymer with more polarized and acidic surface hydroxyls relative to alumina.^54^ Previous work has shown that differences in hydroxyl group density influence the adsorption energetics and interfacial structuring of dodecane through molecular dynamics, with intermediate hydroxyl density promoting detachment of the dodecane from the surface.^55^ Our results showing a higher near-surface PE volume fraction when adsorbed on SiO_2_ are consistent with those findings.

### Polymer Mass and Identity Effects

To understand the effects of polymer molar mass on the adsorbed PE layer, we examined adsorption of two additional molar masses of PE on alumina at 160 °C (10 kg/mol and 207 kg/mol, Figures S7 and S8, respectively). To better illustrate these differences, the total mass of adsorbed polymer was quantified by the first moment of the concentration profile, which is expressed as an effective adsorbed layer thickness, *h*_ads_, defined as

$$h_{ads}=\int_{z=0}^{\infty }\left(\phi_{P}\left(z\right)-\phi_{p}^{bulk}\right)dz$$

where ϕ_p_(*z*) is the volume fraction of the polymer at distance *z* from the substrate and ϕ_p_^bulk^ is the bulk solution concentration. While this effective layer thickness has no physical meaning in terms of the adsorbed polymer structure, this parameter provides a quantitative measure of the total spatial extent of interfacial enrichment and enables direct comparison across polymer identities, molar masses, and oxide supports. The value of this effective layer thickness is equivalent to compacting all the polymer in excess of the bulk solution concentration near the substrate to a pure (solvent-free) polymer film.

Figure 5 summarizes the *h*_ads_ values for the PE polymers on silica and alumina at 160 °C. The dominant effect of surface chemistry is immediately evident. The *h*_ads_ value further quantifies the above data using 34 kg/mol PE, as *h*_ads_ on silica is approximately 5× *h*_ads_ on alumina (Figure 5a). This large increase reflects both high interfacial occupancy and the extension of the weakly adsorbed layer into the solution phase. Figure 5b also shows clear differences in adsorption with molar mass: for PE adsorption on alumina, *h*_ads_ increases monotonically with molar mass.

**Figure 5:**
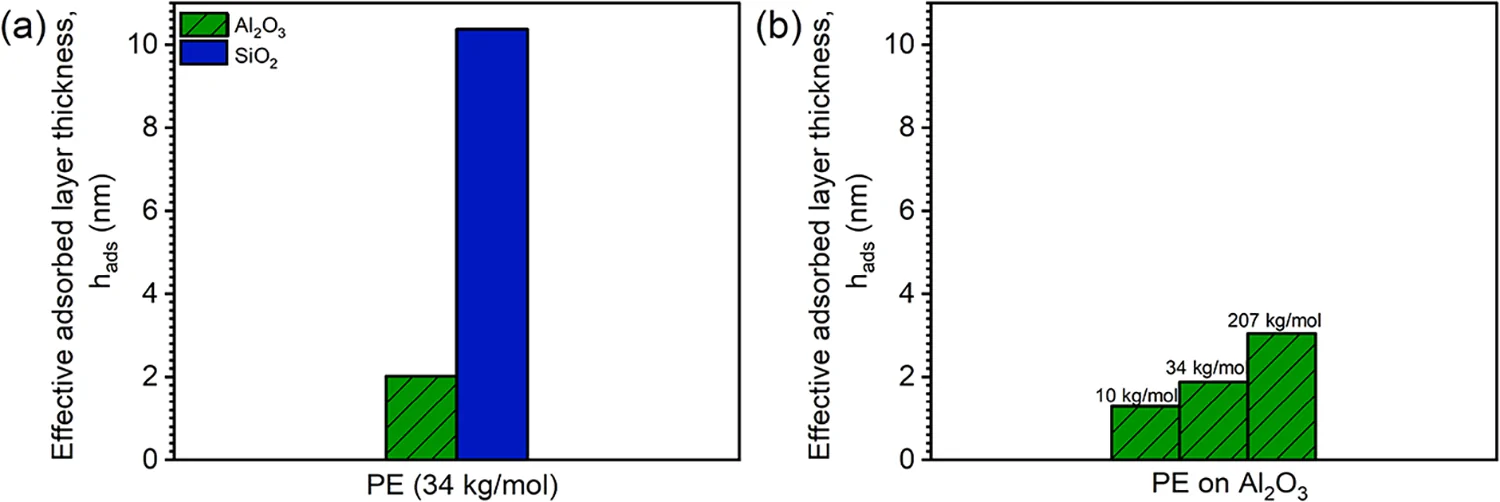
(a) Effective adsorbed layer thickness (*h*_ads_) of 34 kg/mol polyethylene on SiO_2_ and Al_2_O_3_ at 160 °C. (b) Dependence of *h*_ads_ on molar mass for polyethylene adsorbed on Al_2_O_3_ at 160 °C.

The increase in adsorbed effective thickness with molar mass is expected to scale as N^1/2^ from the Scheutjens–Fleer theory for the adsorption of polymers from dilute solution.^56^ If we consider this scaling and the ratios of the effective thickness for the PE adsorbed and different molar masses, the increase in adsorbed quantity of PE with increasing molar mass matches well for 10 to 34 kg/mol, although a slightly larger change in effective thickness would be expected from 34 kg/mol to 207 kg/mol PE (Figure 5b). This difference may be associated with the kinetics of adsorption and lower mobility of the larger molar mass PE (207 kg/mol) to effectively trap a nonequilibrium structure over the time scale associated with the reflectivity measurements (hours). As the structure of adsorbed polymer chains has been shown to evolve over hundreds of hours, it would not be surprising that equilibrium adsorption is not obtained with the higher molar mass PE. Practically, from the perspective of chemical recycling, these slow processes required to achieve equilibrium coverage will likely not be encountered due to the dynamics of the system, where the adsorbed polymers are catalytically broken into smaller fragments.

The surface tensions of PE and iPP are different, which will alter the polymer–surface interactions. Analogous neutron reflectivity measurements and fitting procedures were used to evaluate iPP adsorption on Al_2_O_3_ (Figures S4 and S5) and SiO_2_ (Figure S6) at 160 °C from deuterated dodecane solution. The resulting polymer concentration profiles are shown in Figure 6a. As with PE, the support surface chemistry impacts the adsorption. The adsorption of iPP (23 kg/mol) on SiO_2_ leads to slightly less polymer near the interface than either of the iPP samples adsorbed on Al_2_O_3_ at the same conditions. The differences in the adsorption between silica and alumina are substantially attenuated for iPP in comparison to PE. The methyl branch along the iPP backbone reduces conformational flexibility relative to the linear polyethylene chain and potentially limits efficient segment–surface contact; this could explain the much weaker adsorption on SiO_2_ compared to PE, but the differences in surface tension between PE and iPP may also impact this behavior.

**Figure 6:**
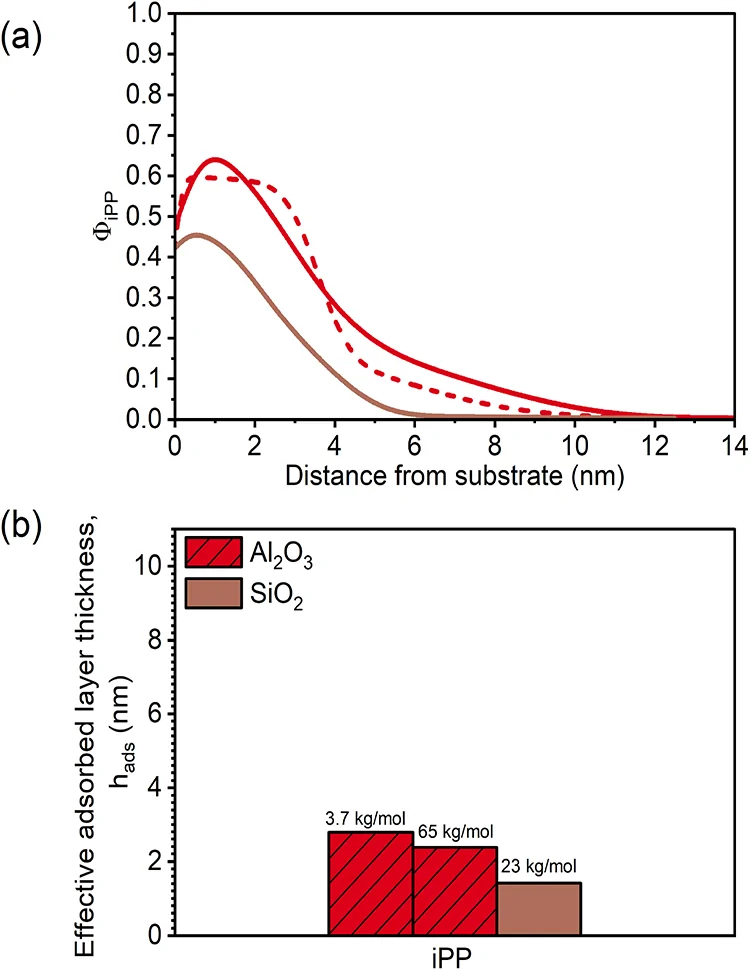
(a) Polypropylene volume fraction (Φ_iPP_) profiles as a function of distance from the substrate for Al_2_O_3_ (red line, 3.7 kg/mol; dotted line, 65 kg/mol) and SiO_2_ (brown line, 23 kg/mol) at 160 °C. The near-surface maximum in Φ_iPP_ arises from convolution with interfacial roughness. (b) Effective adsorbed layer thickness (*h*_ads_) of polypropylene on SiO_2_ (brown) and Al_2_O_3_ (red) at 160 °C.

Two iPP samples of different molar masses (3.7 kg/mol and 65 kg/mol) were used to measure adsorption on Al_2_O_3_ at 160 °C. Although neither of these iPPs matches the *M*_*n*_ of PE (10.7, 34, or 207 kg/mol), the effective layer thickness resulting from iPP adsorption was less than that observed for PE under the same conditions. Interestingly, the iPP of lower *M*_*n*_ adsorbs slightly more on Al_2_O_3_ than the 65 kg/mol iPP; theory suggests that the quantity of chains adsorbed should increase with molar mass of the polymer as N^1/2^.^56^ However, the adsorption includes both entropic and enthalpic contributions—the adsorption of different molar mass iPP was at constant volume fraction in solution, where the number density of chains in solution decreases as *M*_*n*_ increases.

For iPP, the reduction in the amount (effective layer thickness) adsorbed with increasing molar mass is potentially due to the loss of entropy in solution based on the finite number of chains, but we also need to consider that iPP in alkanes is not athermal. The Flory–Huggins interaction parameter, χ, for decane-iPP has been reported to be approximately 0.65–0.7 between 130 and 170 °C,^57,58^ which indicates that the alkane is a poor solvent for iPP. This poor solvent condition suggests that the decrease in the amount of iPP adsorbed with increasing *M*_*n*_ must be driven by entropy. In contrast, the chain connectivity and multiplicity of interactions (enthalpy) appear to drive adsorption of larger chains in PE from large *n*-alkanes (χ = 0.3, good solution) to oxide surfaces. These results illustrate the trade-offs in enthalpy and entropy associated with adsorption that can change the molecular size dependence. Classical polymer adsorption theory from de Gennes^23^ predicts stronger interfacial enrichment for lower molar mass polymers at fixed mass concentration due to the higher number density of short chains that leads to lower conformational entropy penalty per chain for adsorption.

When considering the Al_2_O_3_ surface, the role of the molar mass on the adsorption is readily resolved. For iPP adsorbed on Al_2_O_3,_ increasing molar mass from 3.7 to 65 kg/mol reduces the effective adsorbed thickness (Figure 6b), which is effectively the amount (mass) of polymer adsorbed. This is consistent with classical adsorption arguments in which shorter chains, present at a higher number density and subject to lower conformational entropy penalty per chain, more effectively populate the interface. In contrast, PE on Al_2_O_3_ exhibits an increase in *h*_ads_ with molar mass. This suggests that enhanced segmental contact and an increased number of potential surface interaction points more than offset the entropic penalties associated with adsorbing higher molar mass chains. Across comparable conditions on Al_2_O_3_, polyethylene consistently exhibits a greater effective thickness than isotactic polypropylene, highlighting the influence of chain architecture. The most pronounced adsorption occurs for PE on SiO_2_, which combines favorable surface interactions with the efficient packing of linear chains.

### Implications for Plastic Upcycling

These results have several important implications for catalytic plastic upcycling. Although metal nanoparticles dispersed on oxide supports such as alumina and silica are the active sites for hydrogenolysis^22^ and related C–C bond scission reactions,^10^ factors that influence polymer adsorption on the support govern polymer residence near catalytic sites. The planar oxide films employed here represent simplified model catalyst supports and do not capture the full complexity of practical catalysts that contain nanoscale curvature, porosity, surface defects, and dispersed metal nanoparticles. The curvature is not expected to dramatically impact the polymer adsorption,^52^ but the increased area from the surface roughness of the particles could drive additional adsorption of polymer chains. Nevertheless, the well-defined model surfaces used in this study enable direct measurement of polymer adsorption independent of catalytic reactions, thereby isolating oxide surface chemistry to examine model polyolefins adsorbed from model alkanes. Catalytic plastic upcycling is far more complex, and this work did not consider the influence of broad molar mass distributions, mixtures of polymers, distribution of liquid products, nor hydrogen gas that is critical to upcycling. These latter two aspects will likely impact the adsorption due to changes in the solvent quality^59^ with the high solubility of H_2_ in alkanes,^60^ leading to a reduction in solvent quality. Additionally, the reactions occur under flow, which can impact the adsorption of polymers due to shear stresses that remove weakly adsorbed chains.^61^ Despite these limitations for direct comparison to catalytic systems, the model systems amenable to neutron reflectivity presented here provide some fundamental insights that can be useful for understanding the support effects in catalytic plastic upcycling.

Our results clearly show that differences in oxide support surface chemistry have a dramatic influence on both the structure and size of adsorbed polymer layers. These changes, particularly the density of the adsorbed polymer layer at any given temperature, are critical factors for diffusion of reactants and products to and from catalytically active sites during polymer decomposition reactions. Future work should consider other potential support chemistries to provide deeper insights into the characteristics of the oxide that control the adsorption of polyolefins. From the perspective of plastic upcycling, differences in surface coverage/adsorbed layer density likely impact both the concentration of cleavable bonds available to the catalytically active components as well as surface crowding, which may present significant diffusion barriers for product desorption.

The impact of molar mass on the adsorption of PE on Al_2_O_3_ was small, but higher molar mass PE adsorbed more on the surface, which should promote the deconstruction of larger chains. Real polyolefin waste feeds are likely to contain complex mixtures of polyethylene and polypropylene. Our data show that iPP is less likely to adsorb on either Al_2_O_3_ or SiO_2_ than PE. However, larger iPP polymers may not effectively compete with smaller iPP polymers on the basis of their individual adsorption profiles on Al_2_O_3_. These results likely have important implications, as reaction selectivity governed by polymer adsorption characteristics may be inadvertently attributed to differences in catalytic activity.

Overall, these results establish insights into the control of polyolefin adsorption on oxide surfaces: oxide surface chemistry governs the magnitude of polyolefin adsorption, while molar mass and polymer architecture appear to modulate the detailed structure and spatial extent of the adsorbed layer based on adsorption measurements on Al_2_O_3_ surfaces. These differences in interfacial concentration are likely to influence polymer accessibility to surface-bound catalytic sites and may therefore play a critical role in the support-dependent reactivity and the product distribution in catalytic chemical recycling of polyolefins.

## Conclusion

This work quantifies polyolefin adsorption from model chemical recycling products (dodecane or tetradecane) onto model oxide supports using neutron reflectivity. The use of deuterated alkanes enabled the distinction between polyolefins (polyethylene and isotactic polypropylene) and the solvent as the polymers adsorbed on the surface. Fits of the neutron reflectivity data were used to generate polymer volume fraction profiles and effective adsorbed layer thicknesses for polyethylene and isotactic polypropylene on Al_2_O_3_ and SiO_2_. The adsorption appears to depend strongly on oxide surface chemistry, as considerably more polyethylene adsorbed on silica than alumina. Nearly pure polyethylene was determined to exist within the first ∼5 nm of the SiO_2_ at 160 °C. The adsorption on silica leads to a stratified interfacial structure with a dense oxide-facing layer and a porous solvent-facing layer that could be clearly defined by the fits of the reflectivity data. In contrast, the adsorbed polyethylene layer on Al_2_O_3_ was characterized by a single thin layer within ∼2 nm of the alumina interface. This layer incorporated 20–40% solvent near the oxide surface.

The role of the molar mass on the adsorption depended on the adsorbing polyolefin. For isotactic polypropylene on Al_2_O_3_, increasing the molar mass modestly reduced the amount of polymer adsorbed. Polyethylene exhibited an increase in the adsorbed quantity as its molar mass increased for adsorption on Al_2_O_3_. Across comparable conditions, polyethylene formed thicker adsorbed layers than polypropylene, reflecting differences in the surface interactions and delicate enthalpy and entropy balances that govern the extent of adsorption. Together, these results provide quantitative insights into the behavior of polyolefin adsorption on surfaces that are relevant to catalytic chemical recycling. Since adsorption controls polymer accessibility to surface-bound catalytic sites, the pronounced differences observed between silica and alumina suggest that the surface chemistry of the support provides a route to manipulate reactivity and selectivity in catalytic chemical recycling at higher conversions when appreciable solvent and small molecules are present.

## Supplementary Material



## Notes

SEC elutagrams of synthesized polyethylene and isotactic polypropylene; neutron reflectivity curves, fits, and scattering length density profiles for the bare Al_2_O_3_-coated silicon substrate; and neutron reflectivity curves, fits, and corresponding scattering length density profiles for polyethylene and isotactic polypropylene adsorbed on Al_2_O_3_ and SiO_2_ (PDF)
