# Supplementary material for: Adsorption of Polyolefins from Alkanes on Alumina and Silica
Source: Langmuir. 2026 Oct 5;42(41):30561–71. doi: 10.1021/acs.langmuir.6c03006 (PMC13689828; doi:10.1021/acs.langmuir.6c03006)
Supplement: Supplementary file 1 [file la6c03006_si_001.pdf]

Supporting Information

Adsorption of Polyolefins from Alkanes on Alumina and Silica

Fawaz Motolani,[1] Mason D. Jones,[2] Dayrl Briggs,[3] Bert D. Chandler,[1,4] Brian K. Long,[2] Candice Halbert,[2,3] S. Michael Kilbey II,[2] and Bryan D. Vogt[1,]*

[1]Robert V. Waltemeyer Department of Chemical Engineering, The Pennsylvania State University, University Park, PA 16802 United States

[2]Department of Chemistry, University of Tennessee, Knoxville, Tennessee 36996, United States

[3]Neutron Scattering Division, Oak Ridge National Laboratory, Oak Ridge, TN 37831 United States

[4]Department of Chemistry, The Pennsylvania State University, University Park, PA 16802 United States

*To whom correspondence should be addressed: bdv5051@psu.edu (B.D.V.)

(a)

MJ-1-106 Printed: Monday, February 16, 2026 17:42:04
C:\Users\Univ of TN\Documents\OmniSEC Data\System 1\MJones\2024-09-28_13;10;42_MJ-1-106_01.vdt

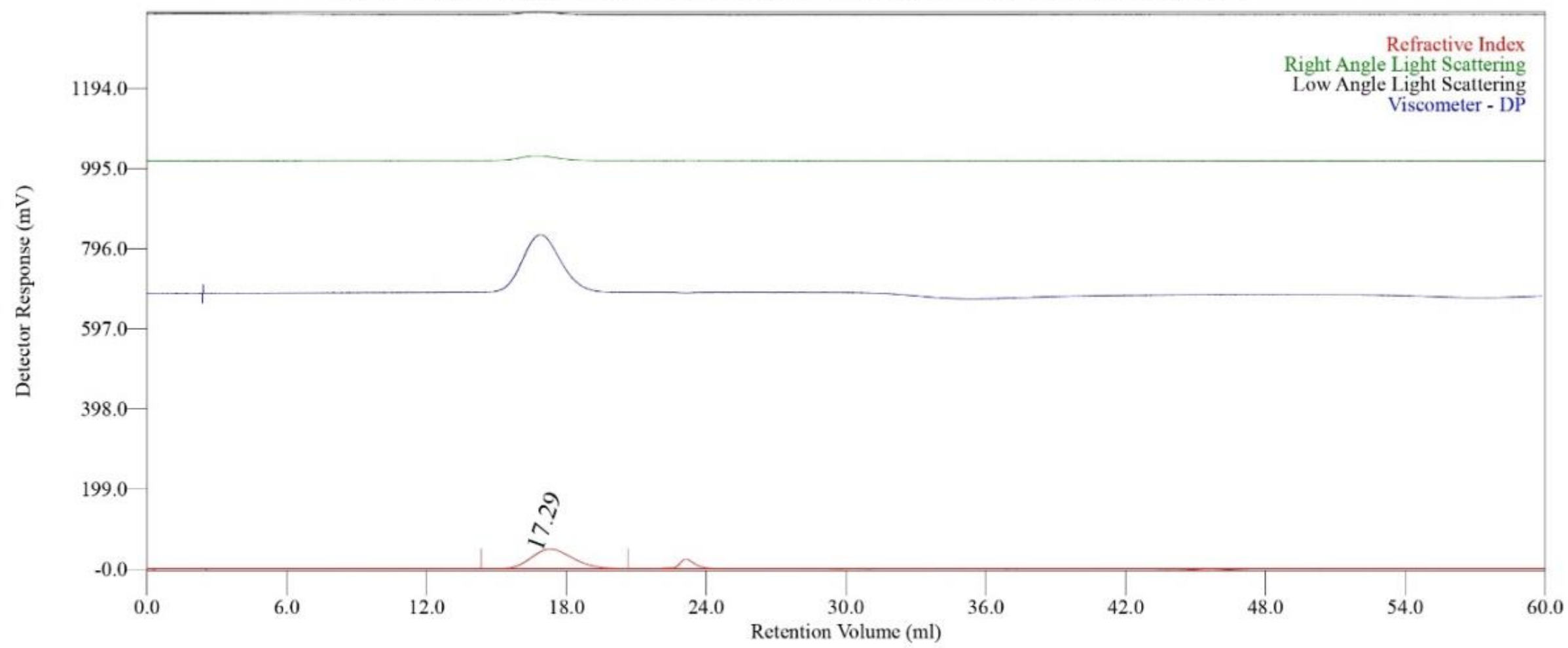

Multi-Detectors - Homopolymers : Results

Peak RV - (ml)	17.293
Mn - (Daltons)	10,919
Mw - (Daltons)	20,153
Mz - (Daltons)	34,425
Mp - (Daltons)	17,317
Mw / Mn	1.846
Percent Above Mw: 0	100.000
Percent Below Mw: 0	0.000
IV - (dl/g)	0.4545
Rh(w) - (nm)	4.994
Rg(w) - (nm)	No Calc
Wt Fr (Peak)	1.000
Mark-Houwink a	0.738
Mark-Houwink logK	-3.496
Branches	0.000
Branch Freq.	0.000
RI Area - (mvml)	102.22
UV Area - (mvml)	0.00
RALS Area - (mvml)	22.27
LALS Area - (mvml)	13.38
IVDP Area - (mvml)	272.33

Sample Parameters	Input	Calculated
Sample Conc - (mg/ml)	4.780	0.000
Sample Recovery (%)	0.000	0.000
dn/dc - (ml/g)	0.0000	0.0901
dA/dc - (ml/g)	0.0000	0.0000

Annotation	
Method File	Long group calibration 09-07-2024-0002.vcm
Limits File	
Date Acquired	Sep 28, 2024 - 13:10:42
Solvent	TCB
Acquisition Operator	admin : Administrator
Calculation Operator	admin : Administrator
Column Set	CLM6210 - HT X 2
System	System 1
Flow Rate - (ml/min)	1.000
Inj Volume - (ul)	200.0
Volume Increment - (ml)	0.00333
Detector Temp. - (deg C)	150.0
Column Temp. - (deg C)	150.0
OmniSEC Build Number	406

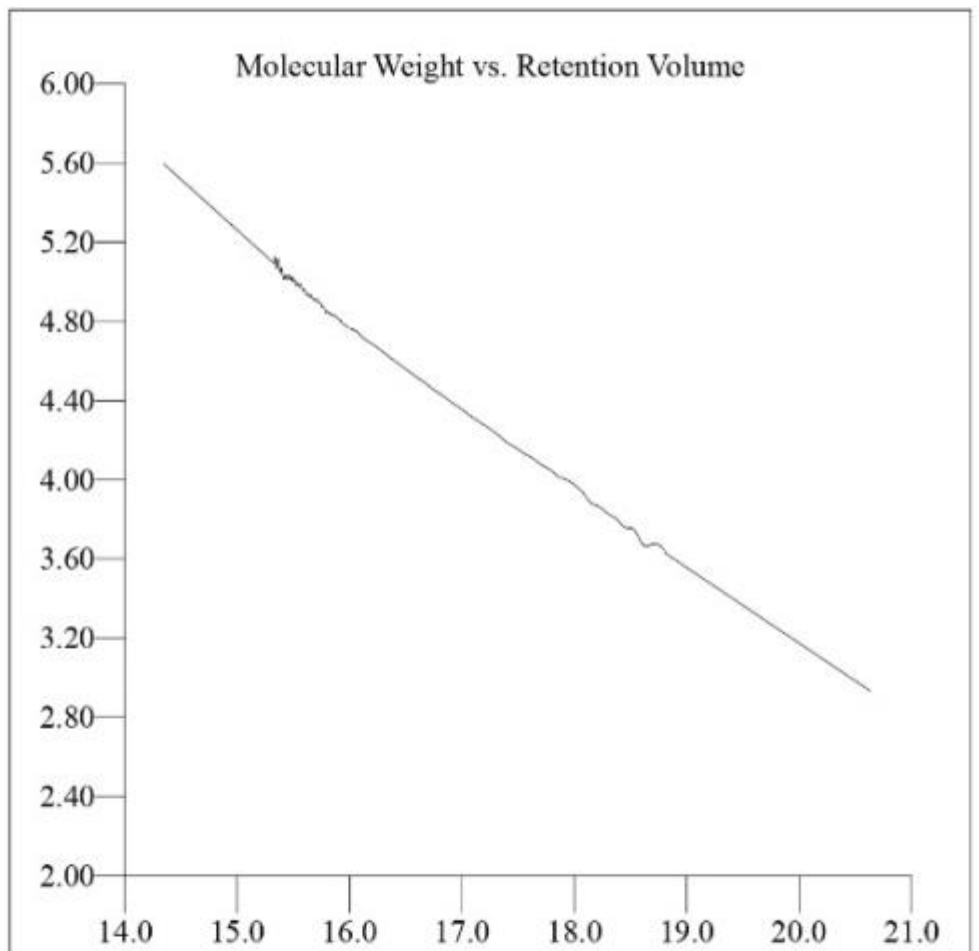

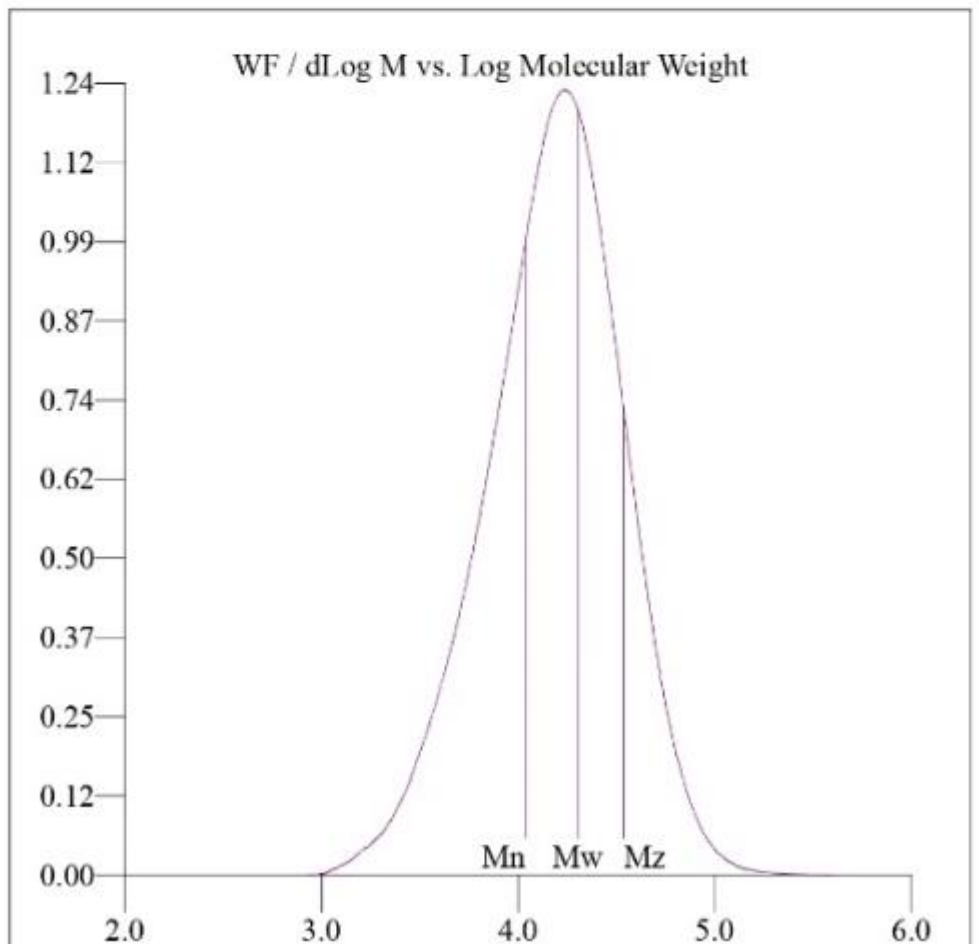

(b)

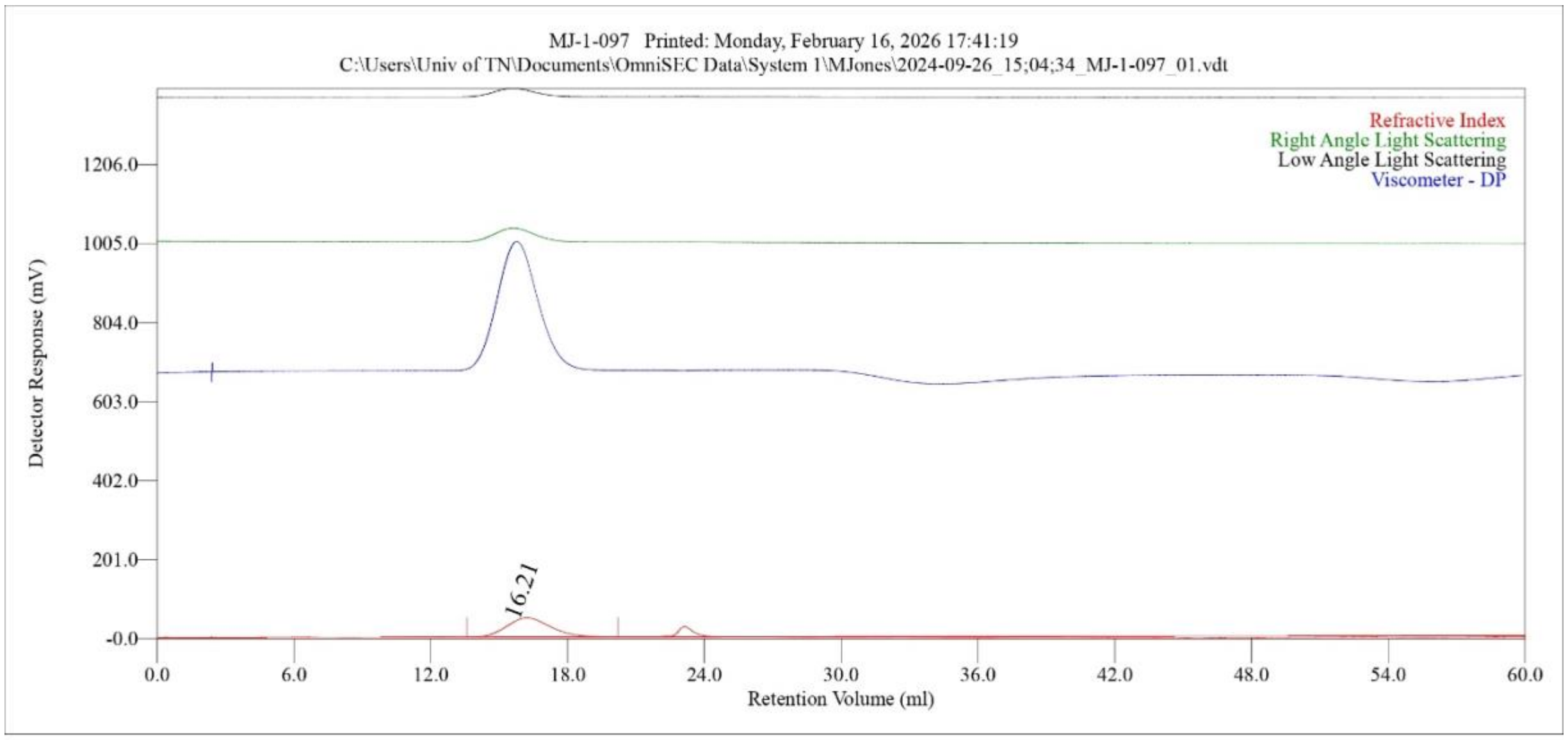

Multi-Detectors - Homopolymers : Results

Peak RV - (ml)	16.207
Mn - (Daltons)	34,789
Mw - (Daltons)	62,824
Mz - (Daltons)	105,574
Mp - (Daltons)	53,396
Mw / Mn	1.806
Percent Above Mw: 0	100.000
Percent Below Mw: 0	0.000
IV - (dl/g)	1.0376
Rh(w) - (nm)	9.606
Rg(w) - (nm)	18.348
Wt Fr (Peak)	1.000
Mark-Houwink a	0.755
Mark-Houwink logK	-3.580
Branches	0.000
Branch Freq.	0.000
RI Area - (mvml)	110.50
UV Area - (mvml)	0.00
RALS Area - (mvml)	72.91
LALS Area - (mvml)	44.90
IVDP Area - (mvml)	679.51

Sample Parameters	Input	Calculated
Sample Conc - (mg/ml)	5.180	0.000
Sample Recovery (%)	0.000	0.000
dn/dc - (ml/g)	0.0000	0.0899
dA/dc - (ml/g)	0.0000	0.0000

Annotation	
Method File	Long group calibration 09-07-2024-0002.vcm
Limits File	
Date Acquired	Sep 26, 2024 - 15:04:34
Solvent	TCB
Acquisition Operator	admin : Administrator
Calculation Operator	admin : Administrator
Column Set	CLM6210 - HT X 2
System	System 1
Flow Rate - (ml/min)	1.000
Inj Volume - (ul)	200.0
Volume Increment - (ml)	0.00333
Detector Temp. - (deg C)	150.0
Column Temp. - (deg C)	150.0
OmniSEC Build Number	406

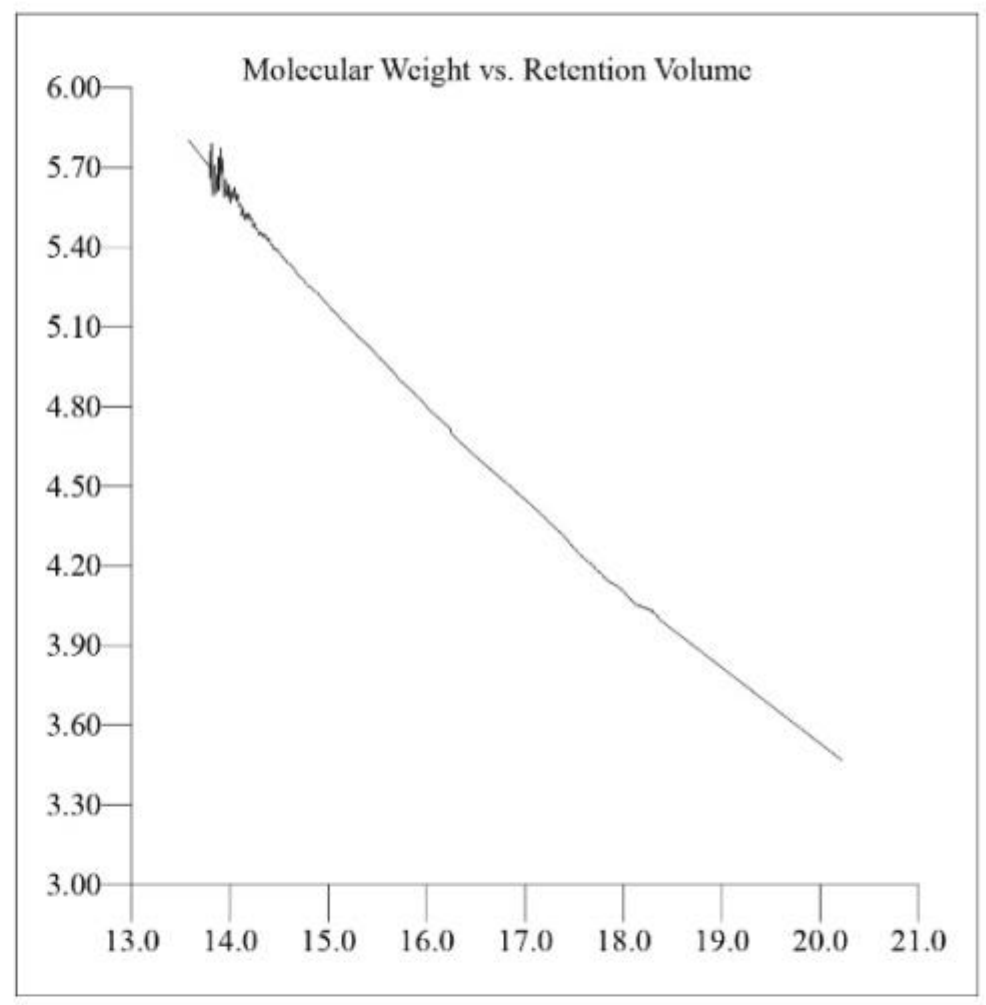

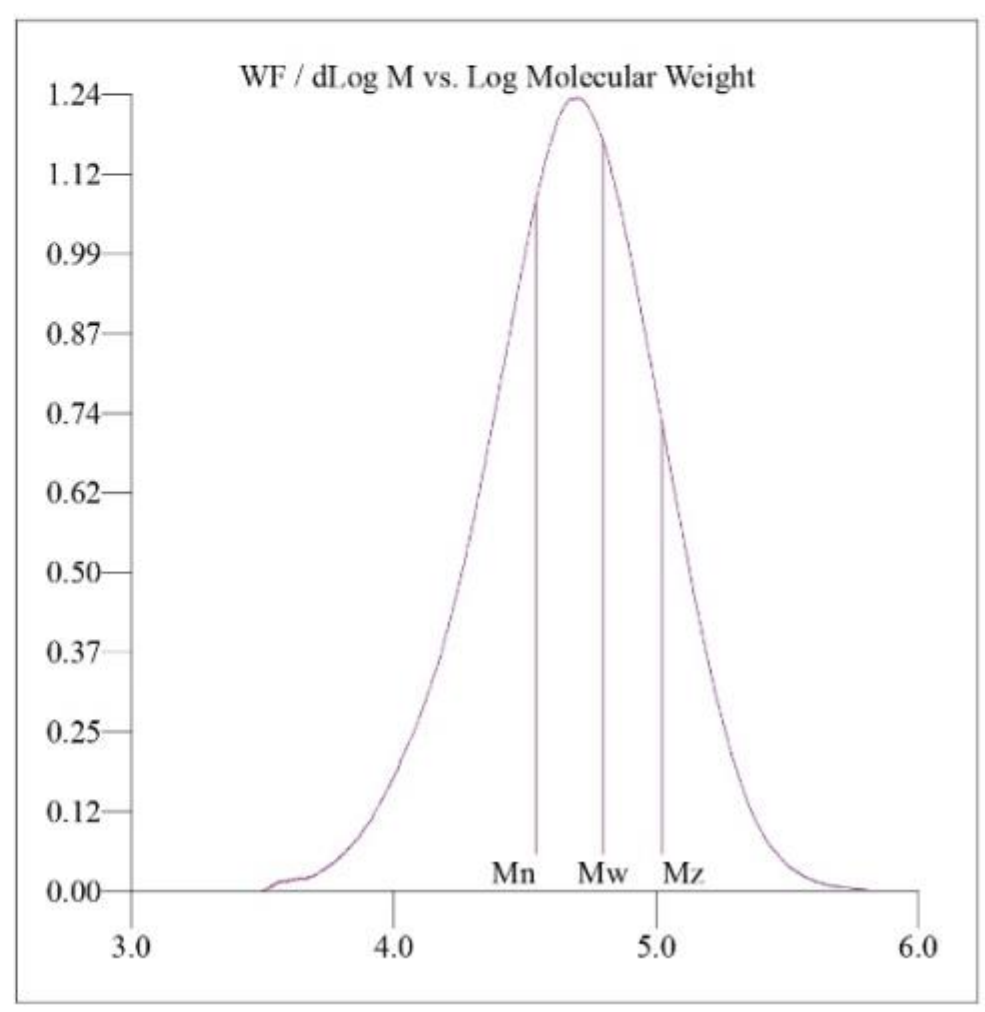

(c)

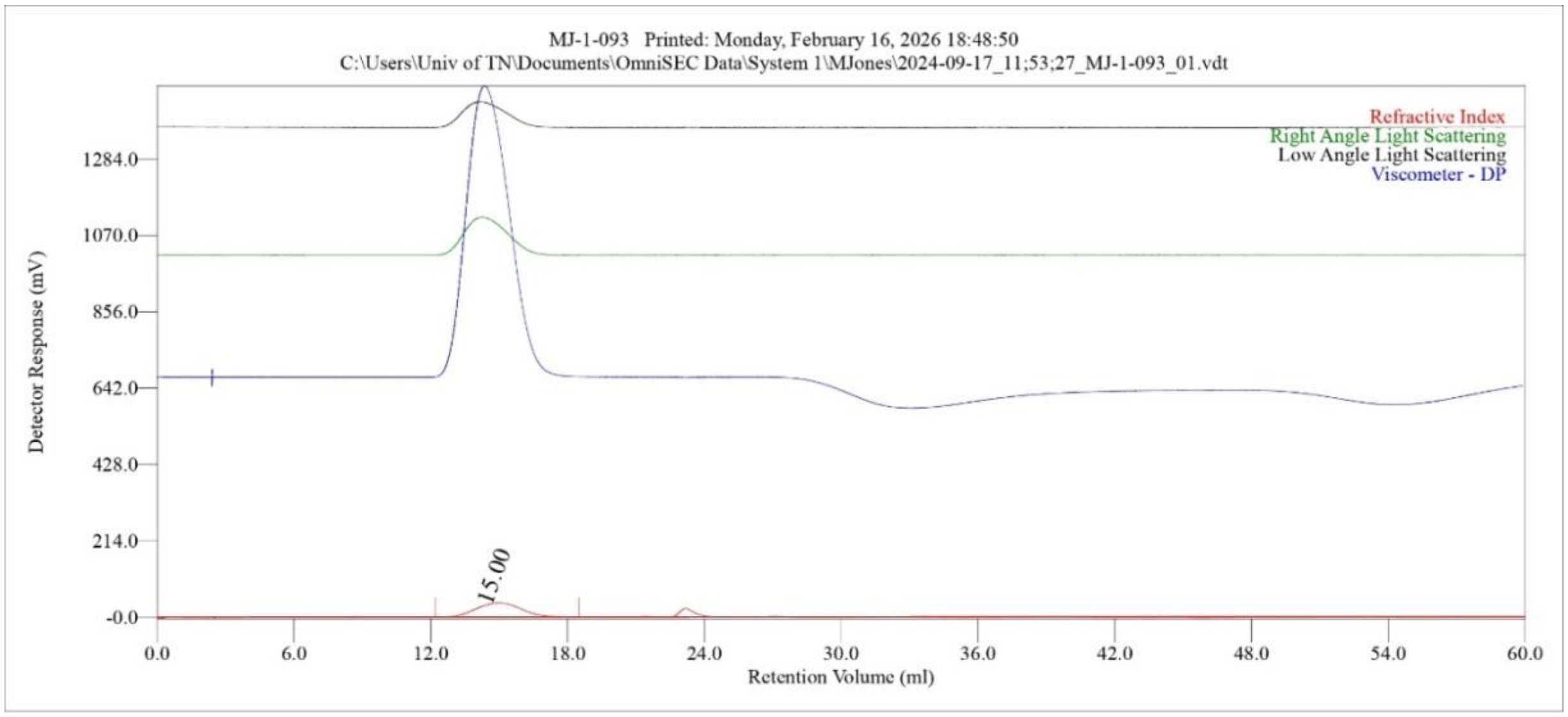

Multi-Detectors - Homopolymers : Results

Peak RV - (ml)	14.997
Mn - (Daltons)	207,080
Mw - (Daltons)	321,811
Mz - (Daltons)	500,001
Mp - (Daltons)	246,060
Mw / Mn	1.554
Percent Above Mw: 0	100.000
Percent Below Mw: 0	0.000
IV - (dl/g)	2.7349
Rh(w) - (nm)	23.105
Rg(w) - (nm)	28.239
Wt Fr (Peak)	1.000
Mark-Houwink a	0.792
Mark-Houwink logK	-3.906
Branches	0.000
Branch Freq.	0.000
RI Area - (mvml)	91.07
UV Area - (mvml)	0.00
RALS Area - (mvml)	233.44
LALS Area - (mvml)	158.92
IVDP Area - (mvml)	1771.50

Sample Parameters	Input	Calculated
Sample Conc - (mg/ml)	5.040	0.000
Sample Recovery (%)	0.000	0.000
dn/dc - (ml/g)	0.0000	0.0761
dA/dc - (ml/g)	0.0000	0.0000

Annotation	
Method File	Long group calibration 09-07-2024-0002.vcm
Limits File	
Date Acquired	Sep 17, 2024 - 11:53:27
Solvent	TCB
Acquisition Operator	admin : Administrator
Calculation Operator	admin : Administrator
Column Set	CLM6210 - HT X 2
System	System 1
Flow Rate - (ml/min)	1.000
Inj Volume - (ul)	200.0
Volume Increment - (ml)	0.00333
Detector Temp. - (deg C)	150.0
Column Temp. - (deg C)	150.0
OmniSEC Build Number	406

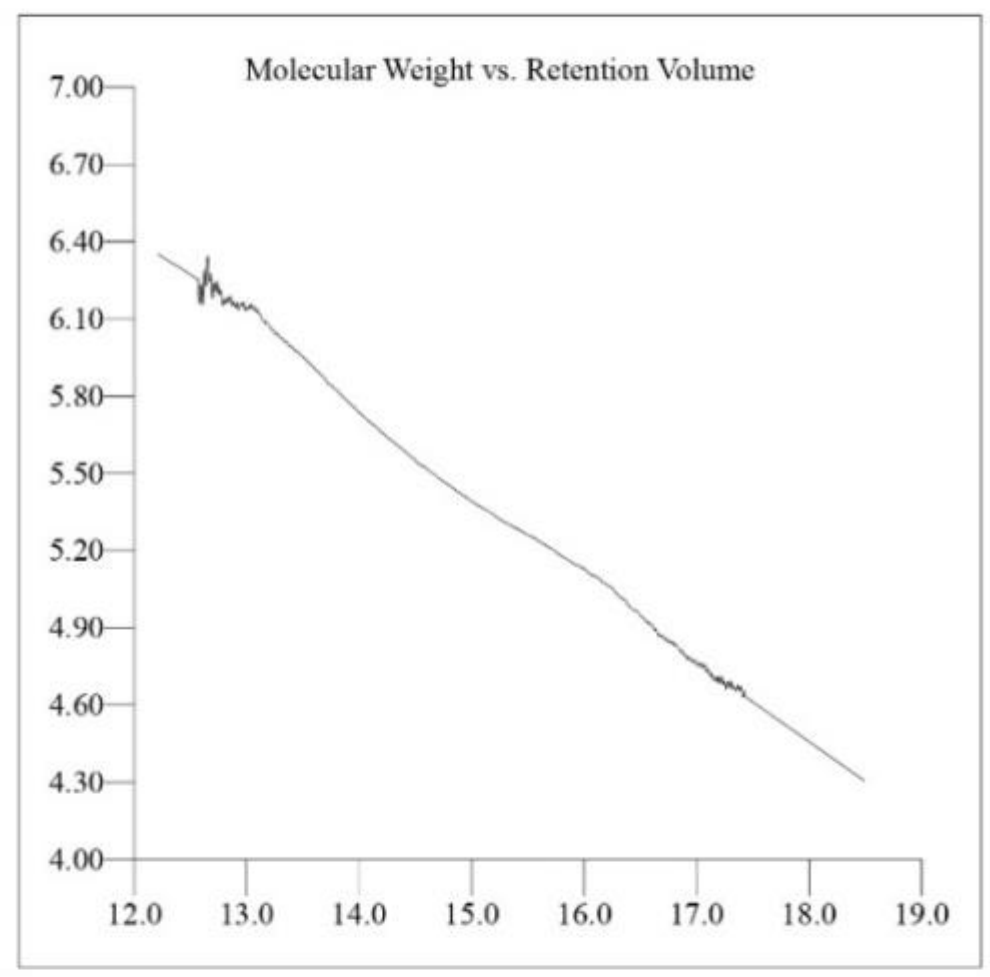

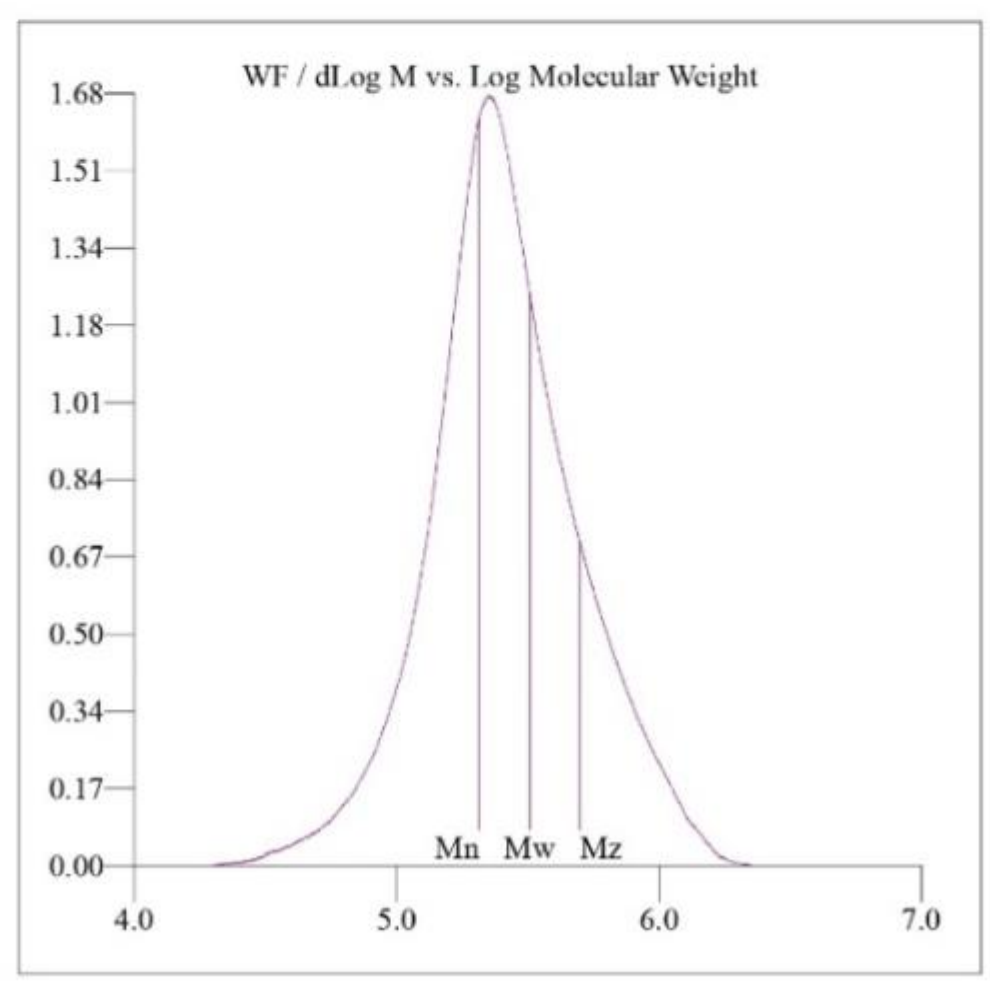

Figure S1. SEC traces and analysis for (a) 10 kg/mol, (b) 34 kg/mol, and (c) 207 kg/mol PE.

(a)

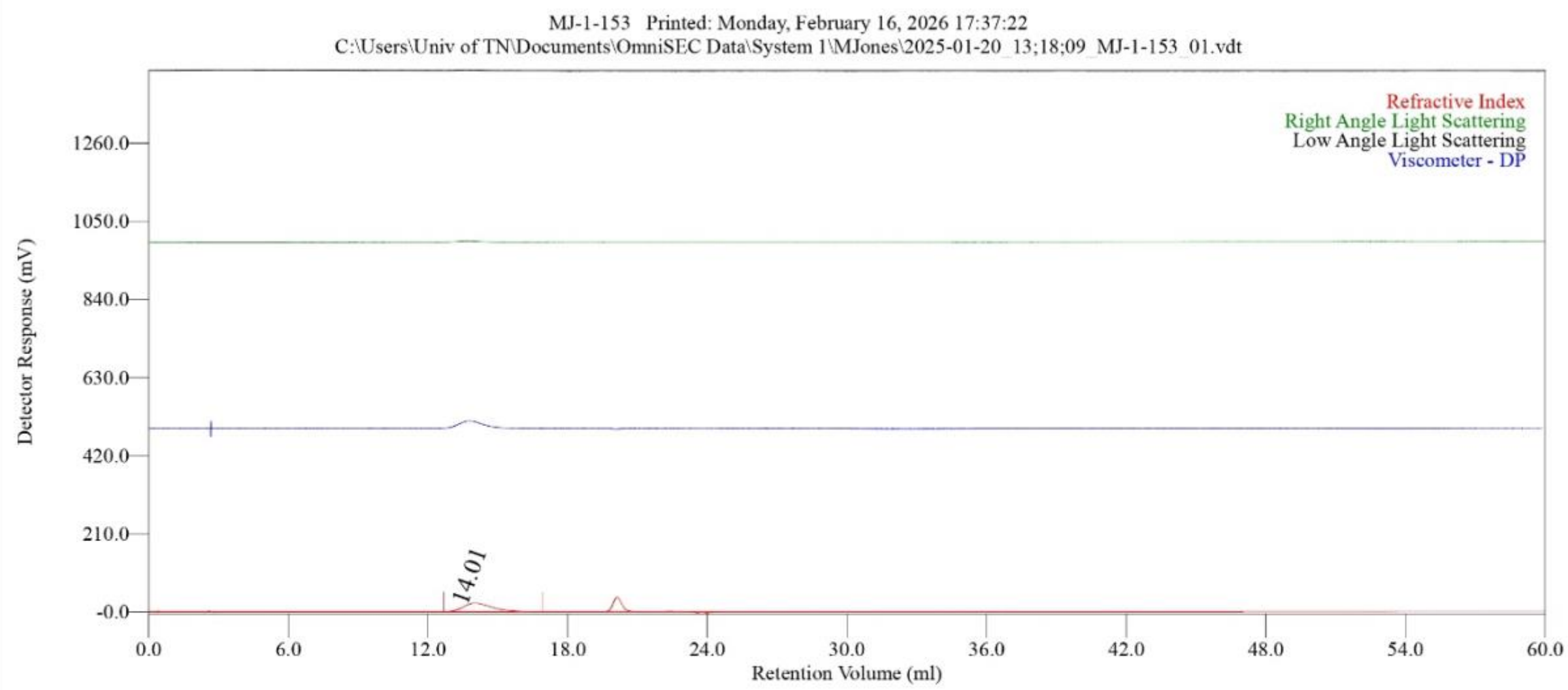

Multi-Detectors - Homopolymers : Results

Peak RV - (ml)		14.010
Mn - (Daltons)		3,712
Mw - (Daltons)		7,827
Mz - (Daltons)		12,730
Mp - (Daltons)		7,693
Mw / Mn		2.109
Percent Above Mw:	0	100.000
Percent Below Mw:	0	0.000
IV - (dl/g)		0.1409
Rh(w) - (nm)		2.457
Rg(w) - (nm)		No Calc
Wt Fr (Peak)		1.000
Mark-Houwink a		0.706
Mark-Houwink logK		-3.573
Branches		0.000
Branch Freq.		0.000
RI Area - (mvml)		32.12
UV Area - (mvml)		0.00
RALS Area - (mvml)		3.59
LALS Area - (mvml)		2.20
IVDP Area - (mvml)		24.29

Sample Parameters	Input	Calculated
Sample Conc - (mg/ml)	1.450	0.000
Sample Recovery (%)	0.000	0.000
dn/dc - (ml/g)	0.0000	0.1080
dA/dc - (ml/g)	0.0000	0.0000

Annotation	
Method File	Long group calibration 01-28-26-0010.vcm
Limits File	
Date Acquired	Jan 20, 2025 - 13:18:09
Solvent	TCB
Acquisition Operator	admin : Administrator
Calculation Operator	admin : Administrator
Column Set	CLM6210 - HT X 2
System	System 1
Flow Rate - (ml/min)	1.000
Inj Volume - (ul)	200.0
Volume Increment - (ml)	0.00333
Detector Temp. - (deg C)	150.0
Column Temp. - (deg C)	150.0
OmniSEC Build Number	406

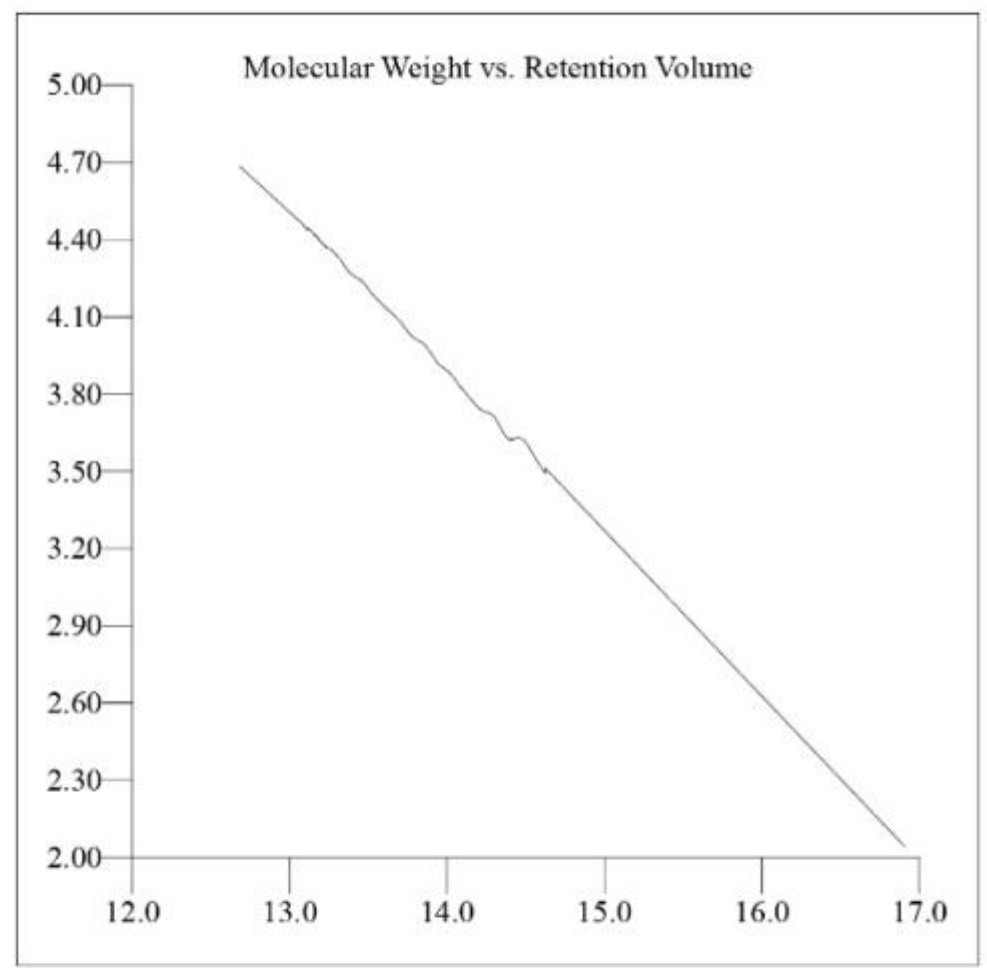

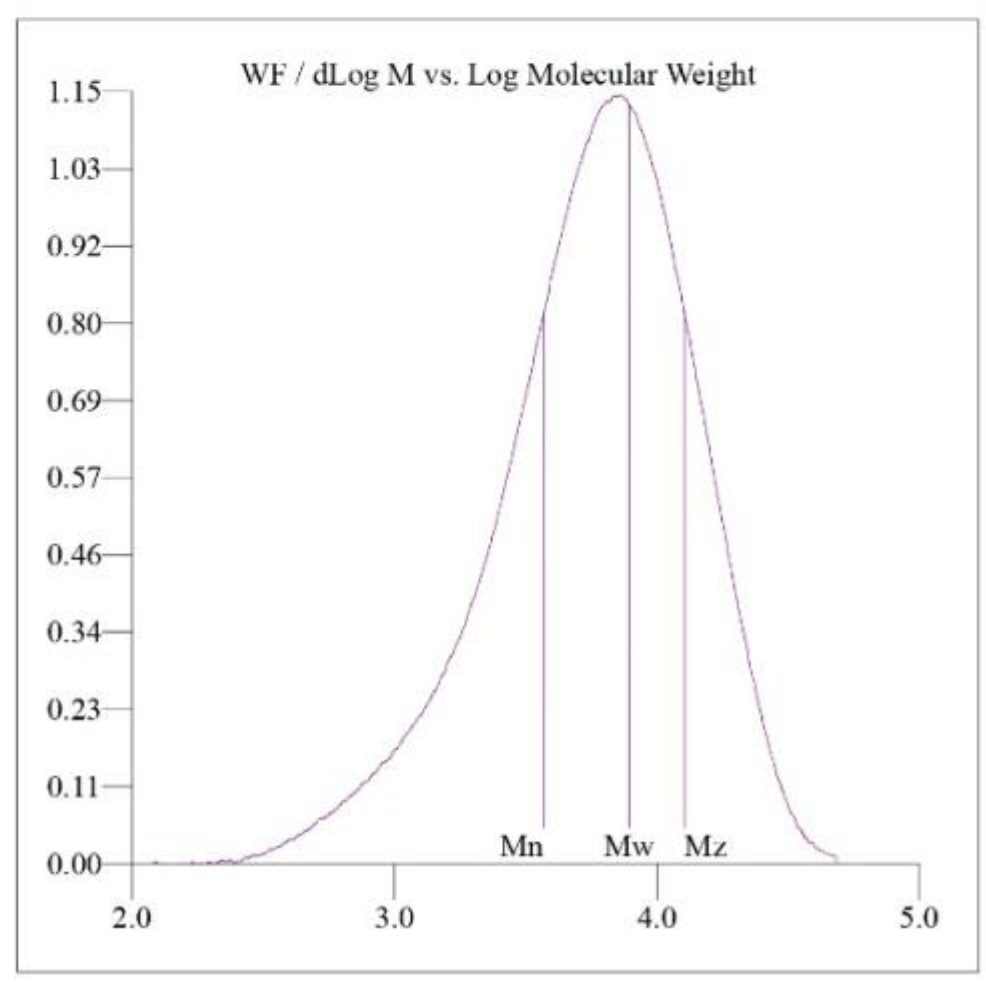

(b)

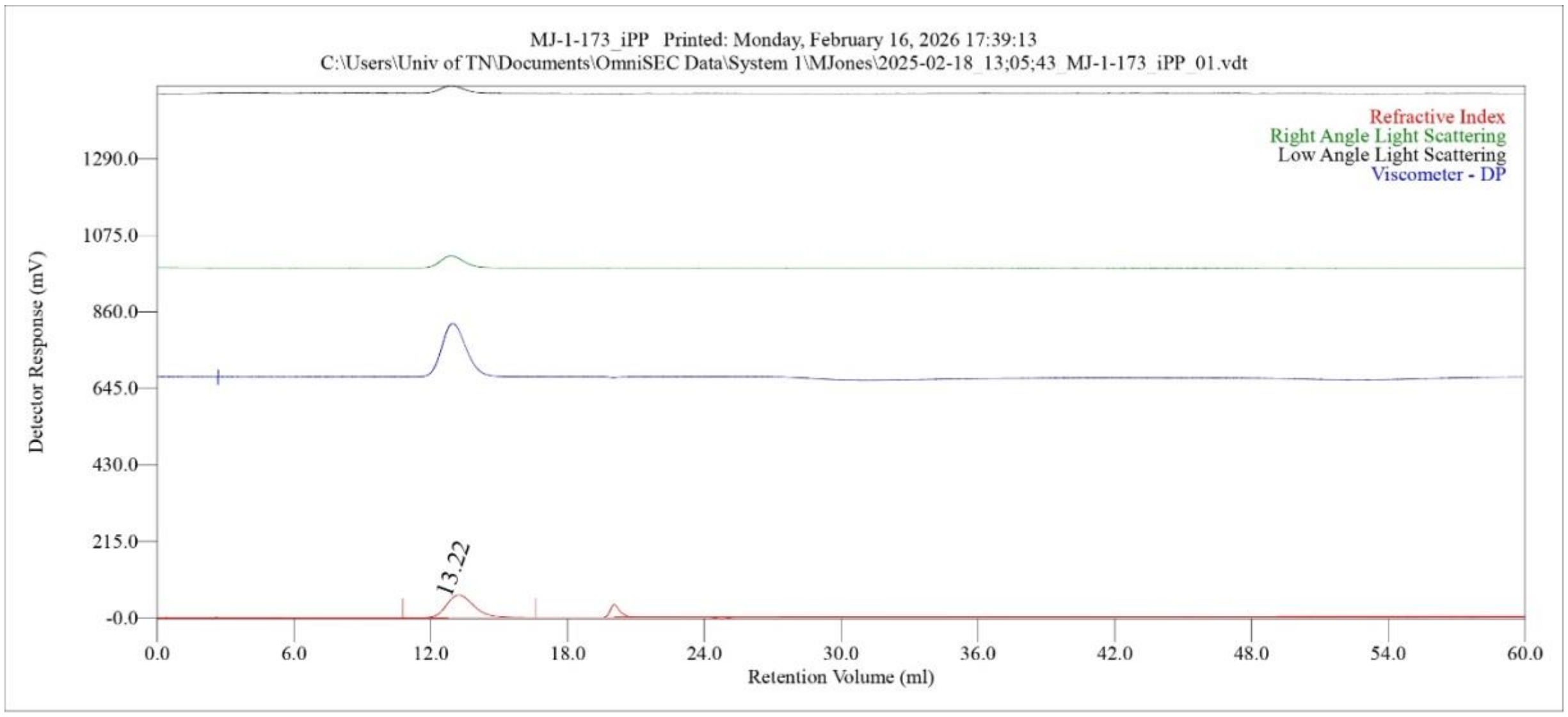

Multi-Detectors - Homopolymers : Results

Peak RV - (ml)	13.217
Mn - (Daltons)	23,171
Mw - (Daltons)	42,115
Mz - (Daltons)	66,902
Mp - (Daltons)	37,425
Mw / Mn	1.817
Percent Above Mw: 0	100.000
Percent Below Mw: 0	0.000
IV - (dl/g)	0.3712
Rh(w) - (nm)	5.992
Rg(w) - (nm)	25.180
Wt Fr (Peak)	1.000
Mark-Houwink a	0.723
Mark-Houwink logK	-3.750
Branches	0.000
Branch Freq.	0.000
RI Area - (mvml)	87.17
UV Area - (mvml)	0.00
RALS Area - (mvml)	39.82
LALS Area - (mvml)	24.88
IVDP Area - (mvml)	184.11

Sample Parameters	Input	Calculated
Sample Conc - (mg/ml)	4.070	0.000
Sample Recovery (%)	0.000	0.000
dn/dc - (ml/g)	0.0000	0.0925
dA/dc - (ml/g)	0.0000	0.0000

Annotation	
Method File	Long group calibration 01-15-2025-updated-0001.vcm
Limits File	
Date Acquired	Feb 18, 2025 - 13:05:43
Solvent	TCB
Acquisition Operator	admin : Administrator
Calculation Operator	admin : Administrator
Column Set	CLM6210 - HT X 2
System	System 1
Flow Rate - (ml/min)	1.000
Inj Volume - (ul)	200.0
Volume Increment - (ml)	0.00333
Detector Temp. - (deg C)	150.0
Column Temp. - (deg C)	150.0
OmniSEC Build Number	406

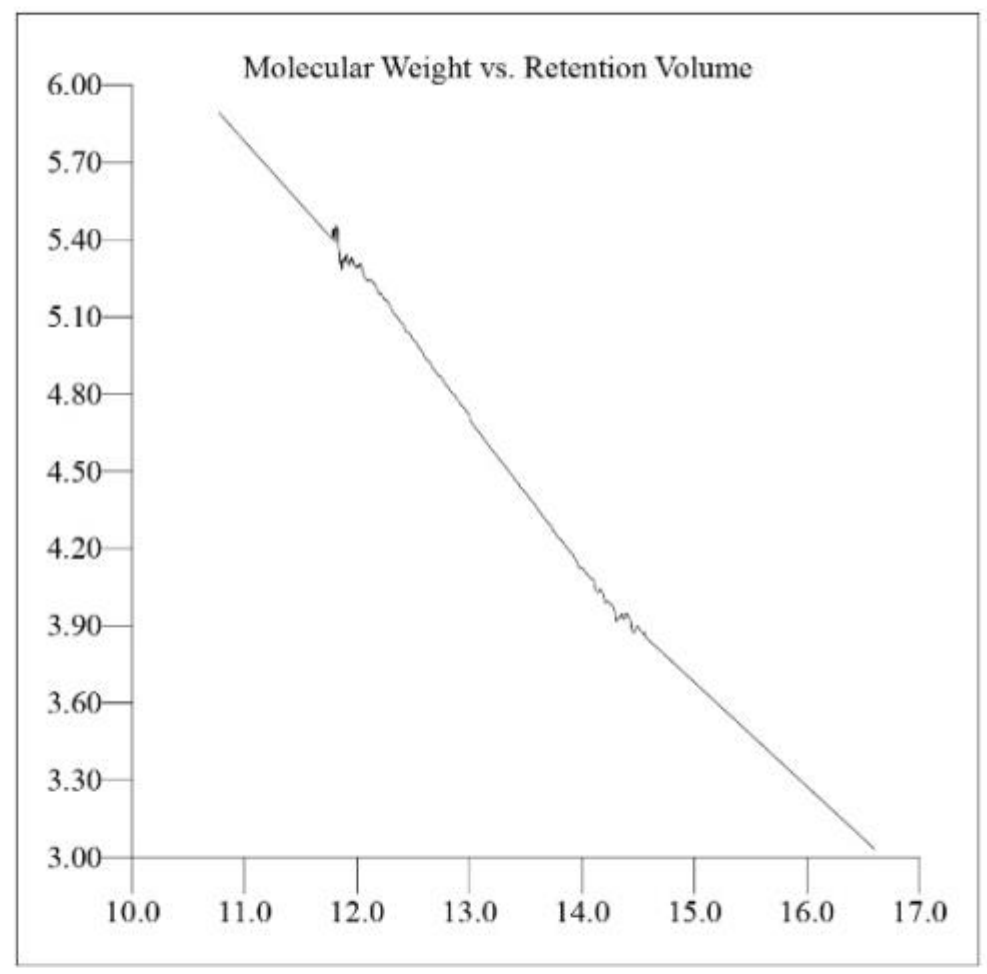

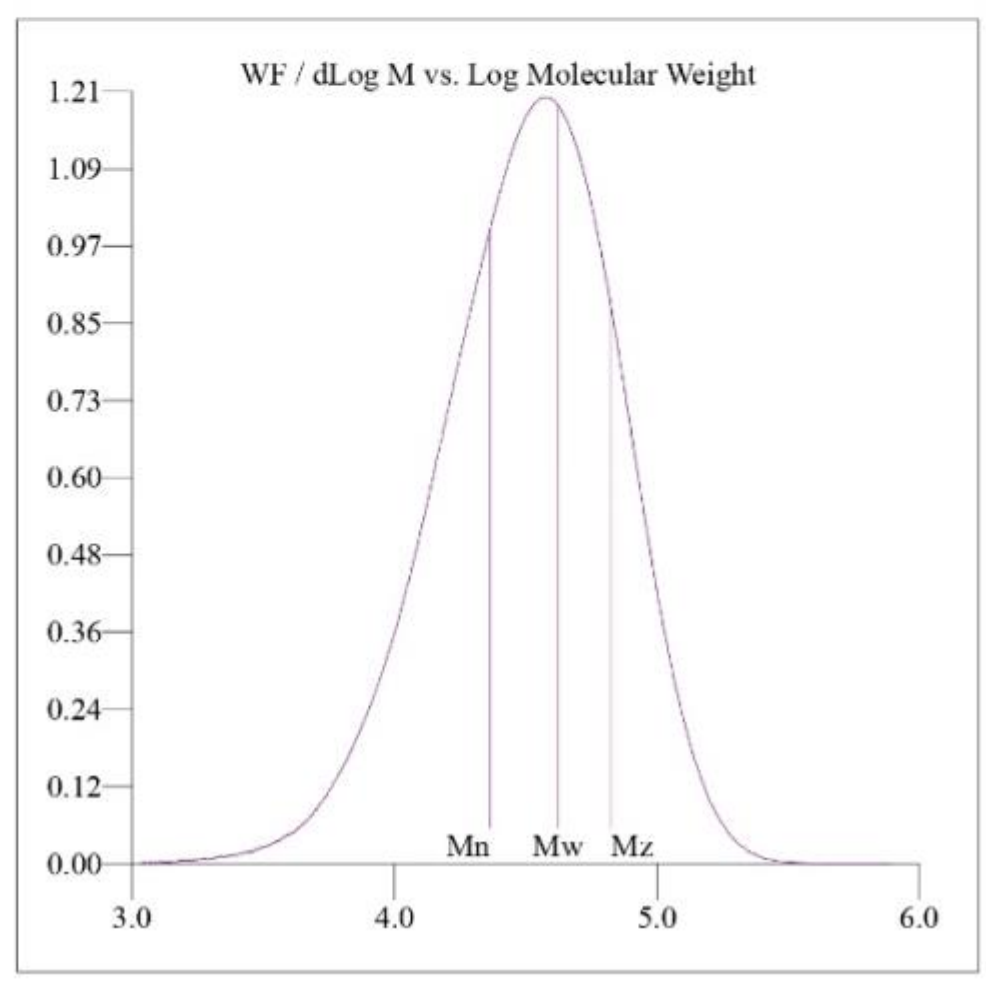

(c)

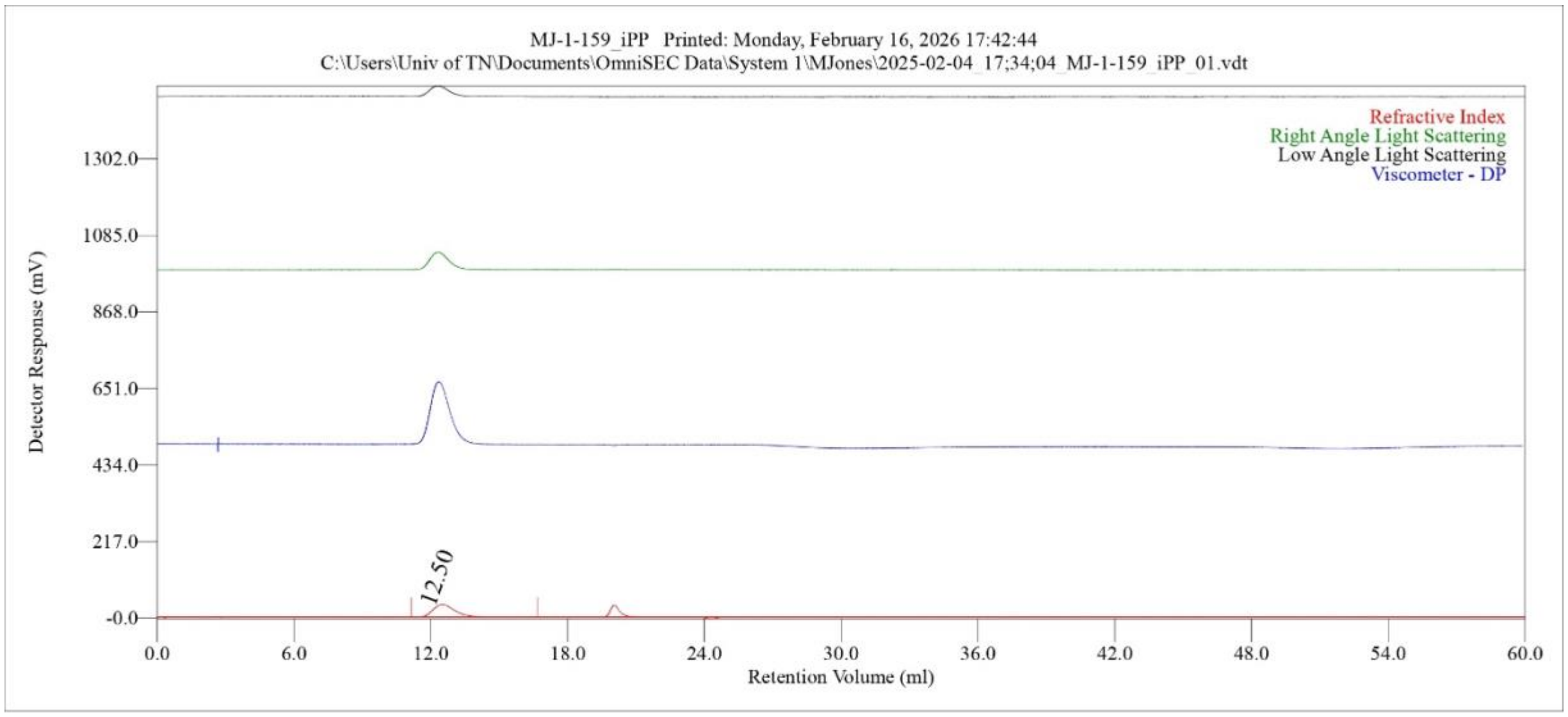

Multi-Detectors - Homopolymers : Results

Peak RV - (ml)	12.500
Mn - (Daltons)	65,130
Mw - (Daltons)	111,798
Mz - (Daltons)	144,799
Mp - (Daltons)	116,973
Mw / Mn	1.717
Percent Above Mw: 0	100.000
Percent Below Mw: 0	0.000
IV - (dl/g)	0.7473
Rh(w) - (nm)	10.655
Rg(w) - (nm)	No Calc
Wt Fr (Peak)	1.000
Mark-Houwink a	0.792
Mark-Houwink logK	-4.111
Branches	0.000
Branch Freq.	0.000
RI Area - (mvml)	40.51
UV Area - (mvml)	0.00
RALS Area - (mvml)	48.21
LALS Area - (mvml)	29.02
IVDP Area - (mvml)	176.97

Sample Parameters	Input	Calculated
Sample Conc - (mg/ml)	1.940	0.000
Sample Recovery (%)	0.000	0.000
dn/dc - (ml/g)	0.0000	0.0902
dA/dc - (ml/g)	0.0000	0.0000

Annotation	
Method File	Long group calibration 01-15-2025-updated-0001.vcm
Limits File	
Date Acquired	Feb 04, 2025 - 17:34:04
Solvent	TCB
Acquisition Operator	admin : Administrator
Calculation Operator	admin : Administrator
Column Set	CLM6210 - HT X 2
System	System 1
Flow Rate - (ml/min)	1.000
Inj Volume - (ul)	200.0
Volume Increment - (ml)	0.00333
Detector Temp. - (deg C)	150.0
Column Temp. - (deg C)	150.0
OmniSEC Build Number	406

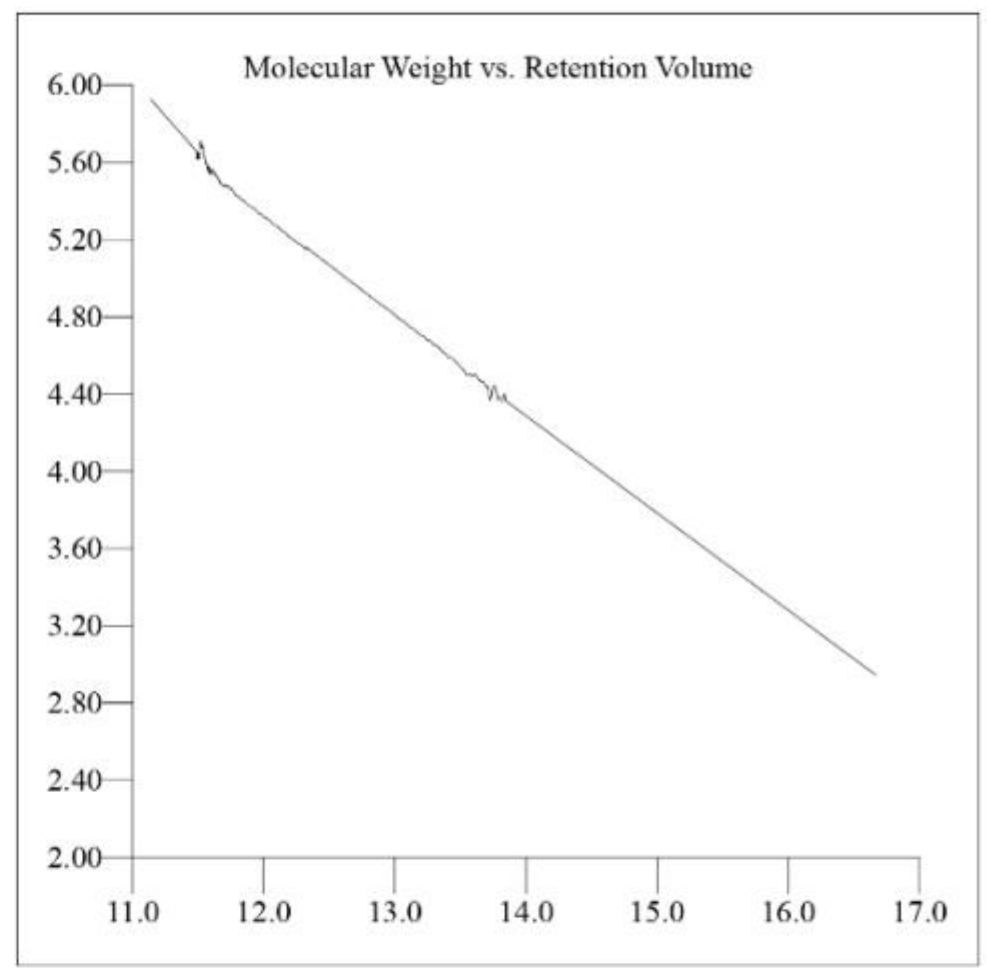

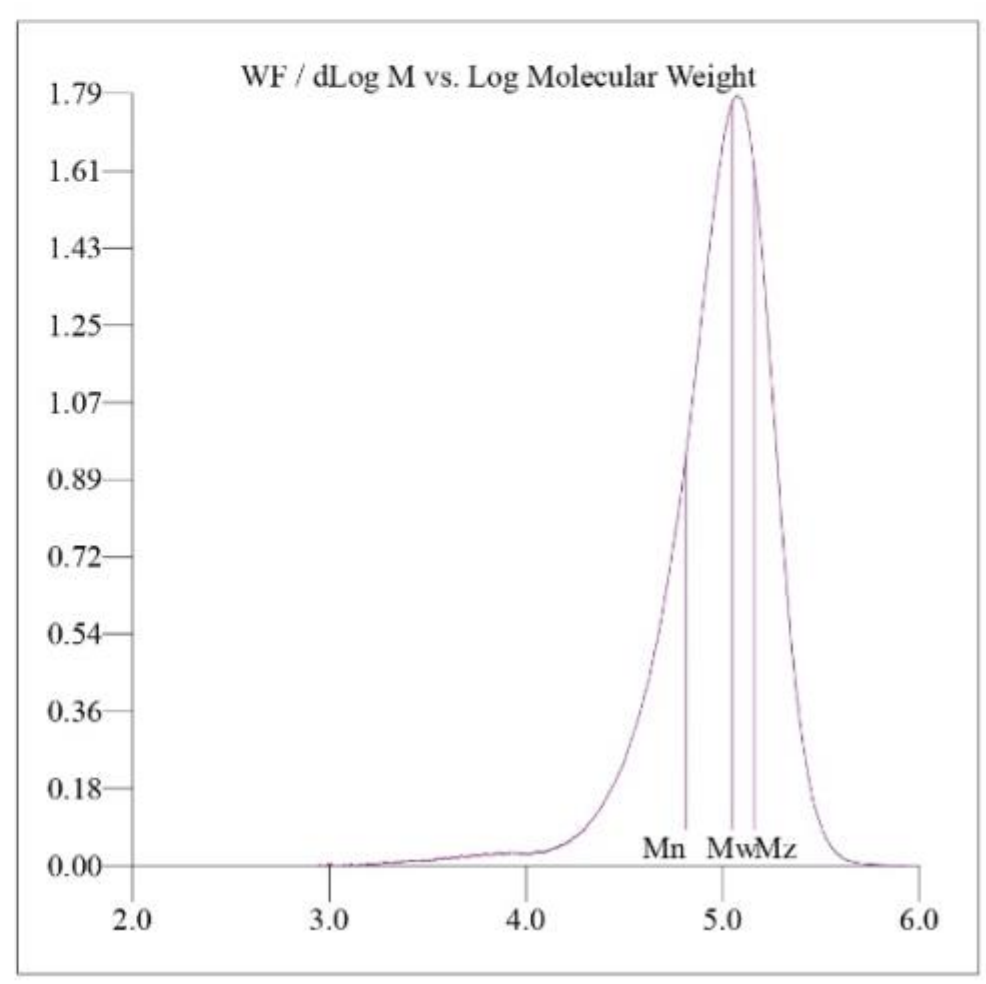

Figure S2. SEC traces and analysis for (a) 3.7 kg/mol, (b) 23 kg/mol, and (c) 65 kg/mol iPP.

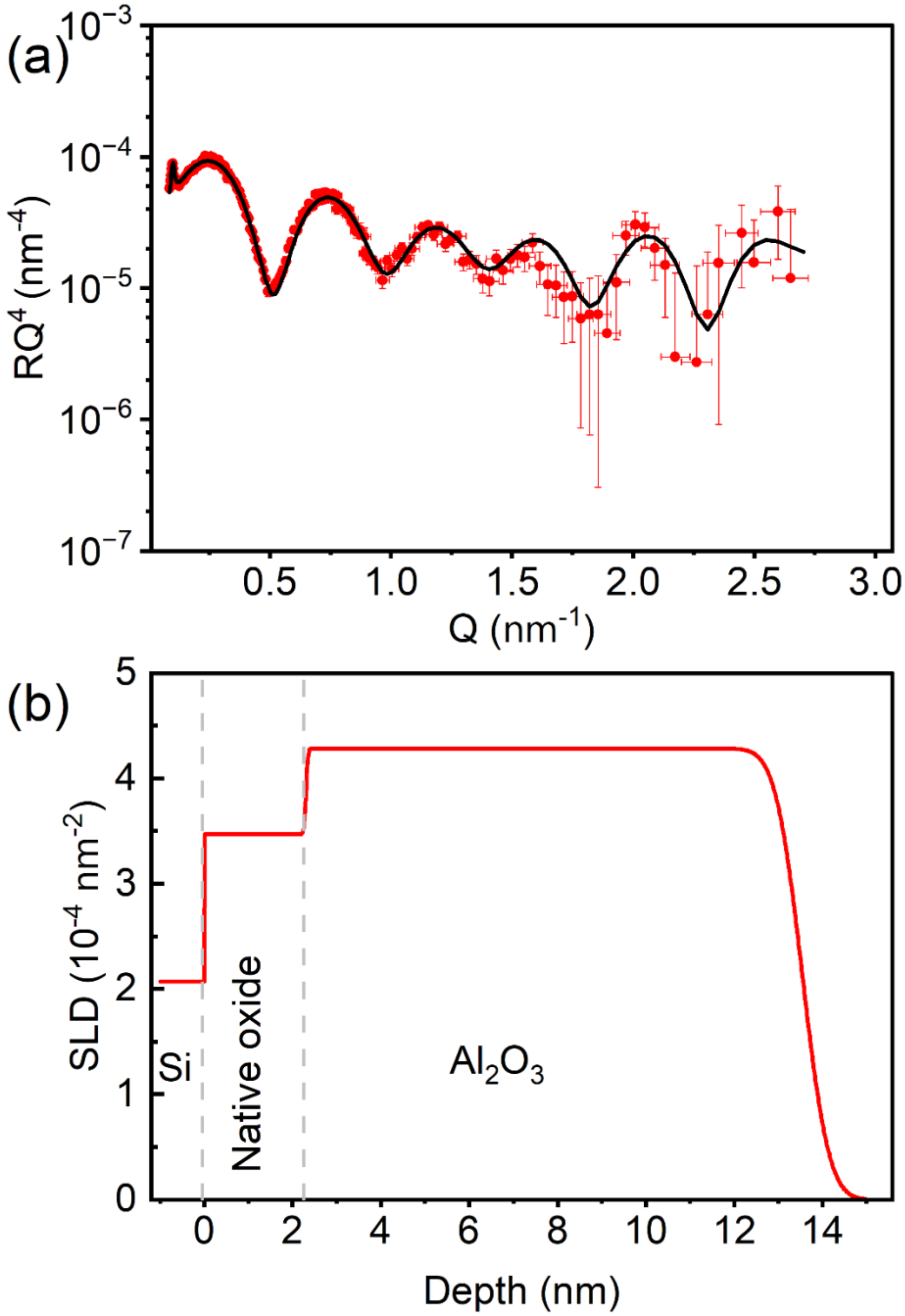

Figure S3. (a) Neutron reflectivity (NR) data (symbols) and model fits (solid lines) of bare Al_2O_3 coated silicon substrate. (b) SLD profiles from the fit showing the layers depth.

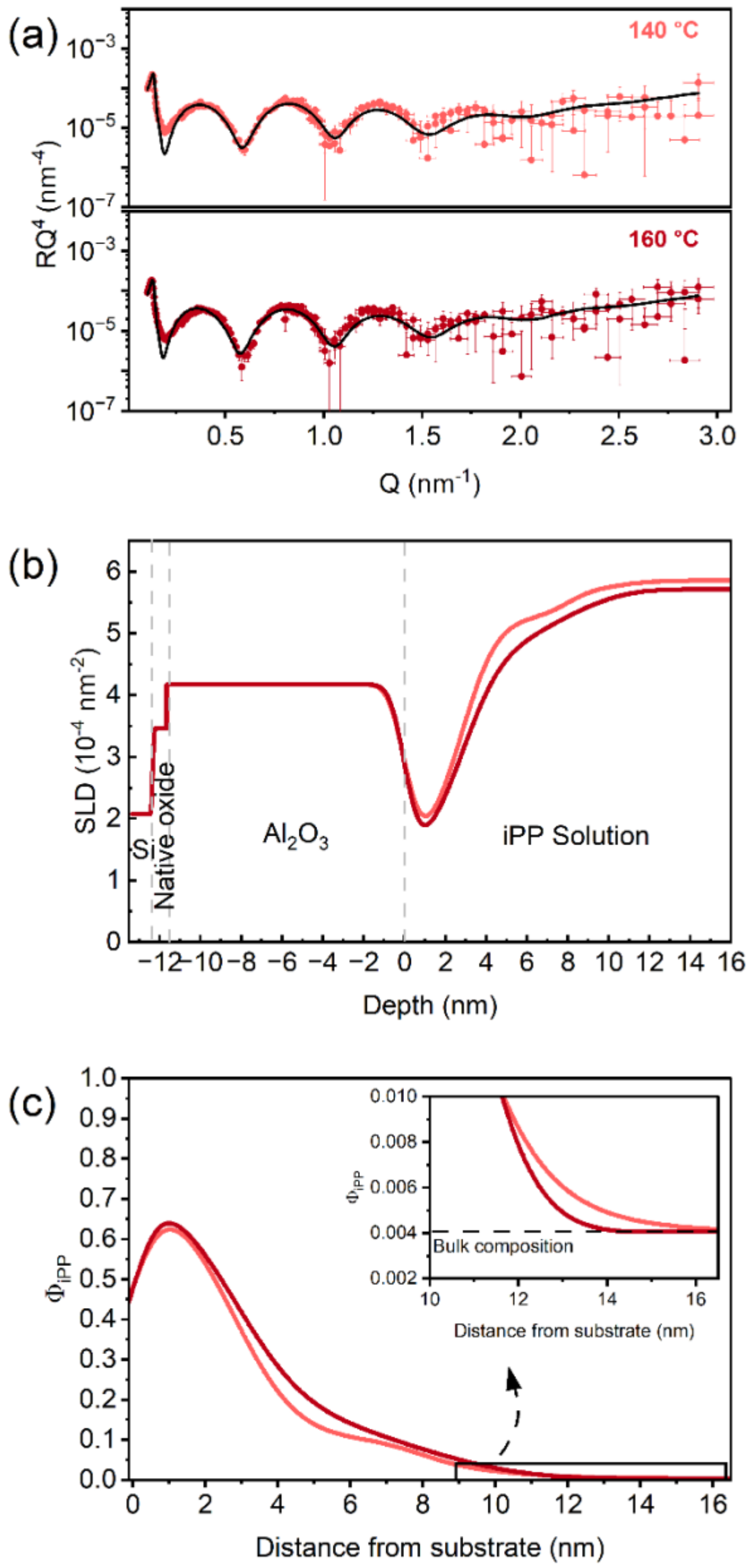

Figure S4. (a) Neutron reflectivity (NR) data (symbols) and model fits (solid lines) for 0.4 wt % 3.7 kg/mol iPP in dC_{12} adsorbed on Al_2O_3 at 140 °C (bright red) and 160 °C (dark red). (b) SLD profiles normal to the interface obtained from the fitted models at 140 °C (bright red) and 160 °C (dark red). (c) Corresponding polypropylene volume fraction (Φ_{iPP}) profiles as a function of distance from the Al_2O_3 surface. The inset highlights the decay of the polymer volume fraction toward the bulk solution.

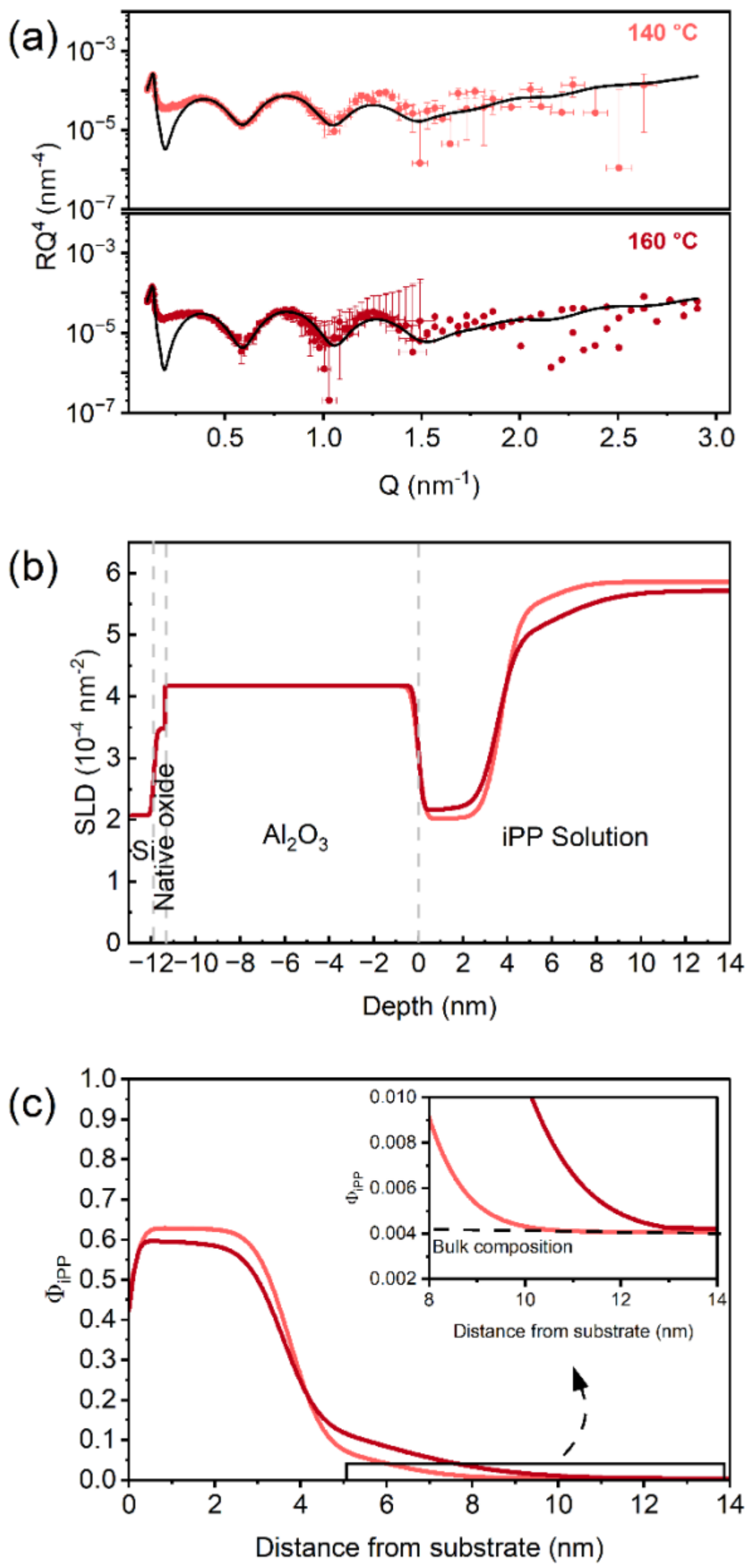

Figure S5. (a) Neutron reflectivity (NR) data (symbols) and model fits (solid lines) for 0.4 wt % 65 kg/mol iPP in dC_{12} adsorbed on Al_2O_3 at 140 °C (bright red) and 160 °C (dark red). (b) SLD profiles normal to the interface obtained from the fitted models at 140 °C (bright red) and 160 °C (dark red). (c) Corresponding polypropylene volume fraction (Φ_{iPP}) profiles as a function of distance from the Al_2O_3 surface. The inset highlights the decay of the polymer volume fraction toward the bulk solution.

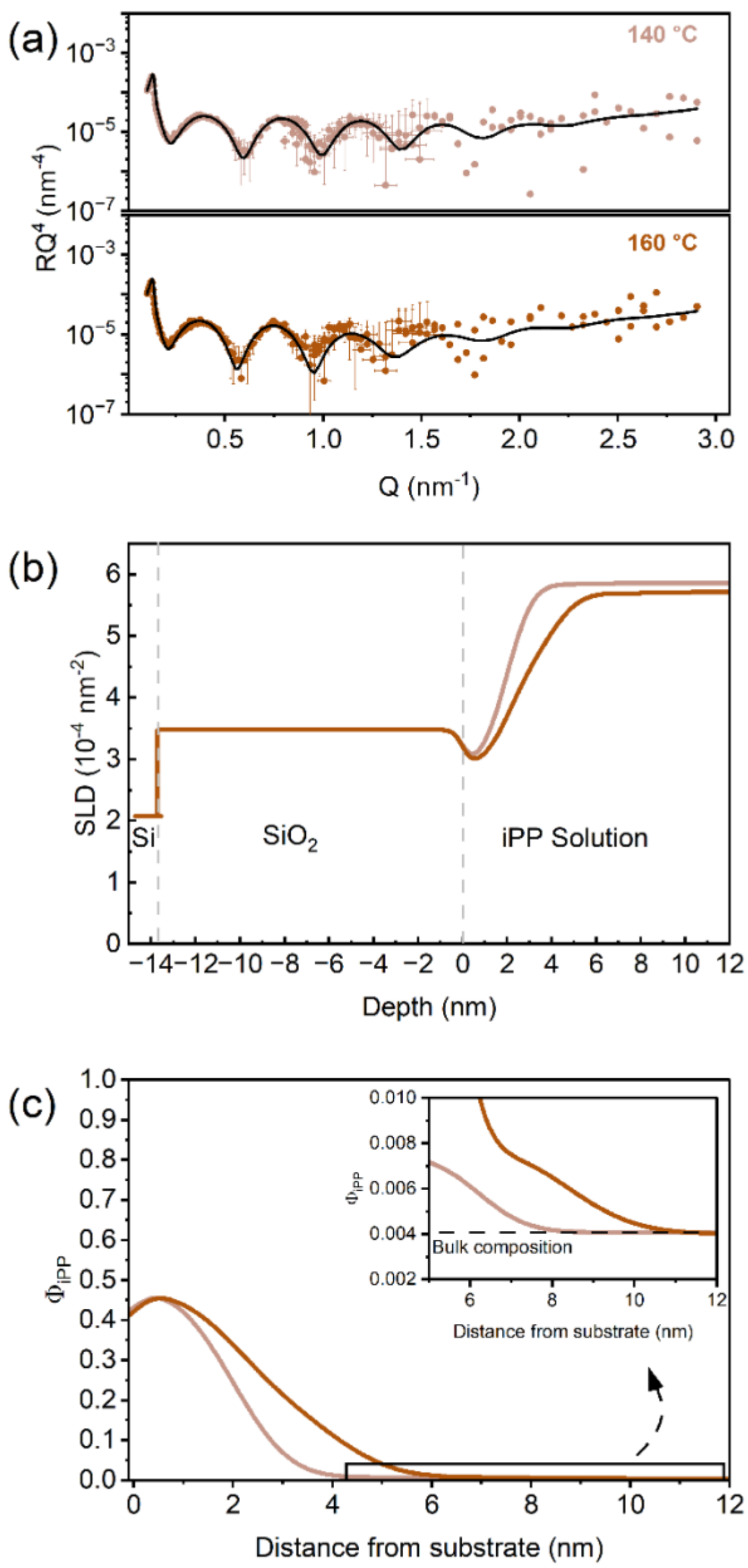

Figure S6. (a) Neutron reflectivity (NR) data (symbols) and model fits (solid lines) for 0.4 wt % 23 kg/mol iPP in dC_{12} adsorbed on SiO_2 at 140 °C (brown) and 160 °C (dark brown). (b) SLD profiles normal to the interface obtained from the fitted models at 140 °C (brown) and 160 °C (dark brown). (c) Corresponding polypropylene volume fraction (Φ_{iPP}) profiles as a function of distance from the SiO_2 surface. The inset highlights the decay of the polymer volume fraction toward the bulk solution.

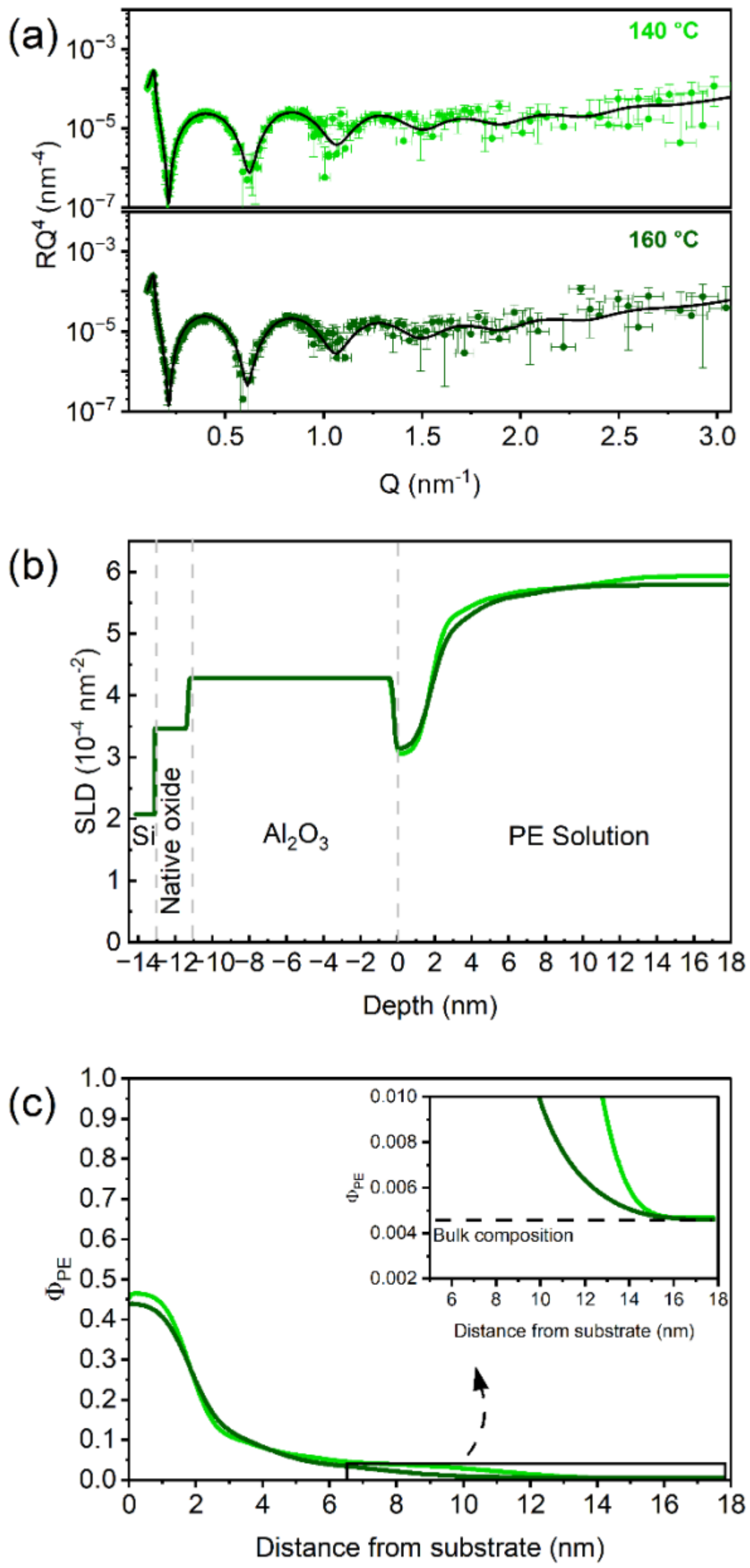

Figure S7. (a) Neutron reflectivity (NR) data (symbols) and model fits (solid lines) for 0.4 wt % 10 kg/mol PE in dC_{14} adsorbed on Al_2O_3 at 140 °C (bright green) and 160 °C (dark green). (b) SLD profiles normal to the interface obtained from the fitted models at 140 °C (bright green) and 160 °C (dark green). (c) Corresponding polyethylene volume fraction (Φ_{PE}) profiles as a function of distance from the Al_2O_3 surface. The inset highlights the decay of the polymer volume fraction toward the bulk solution.

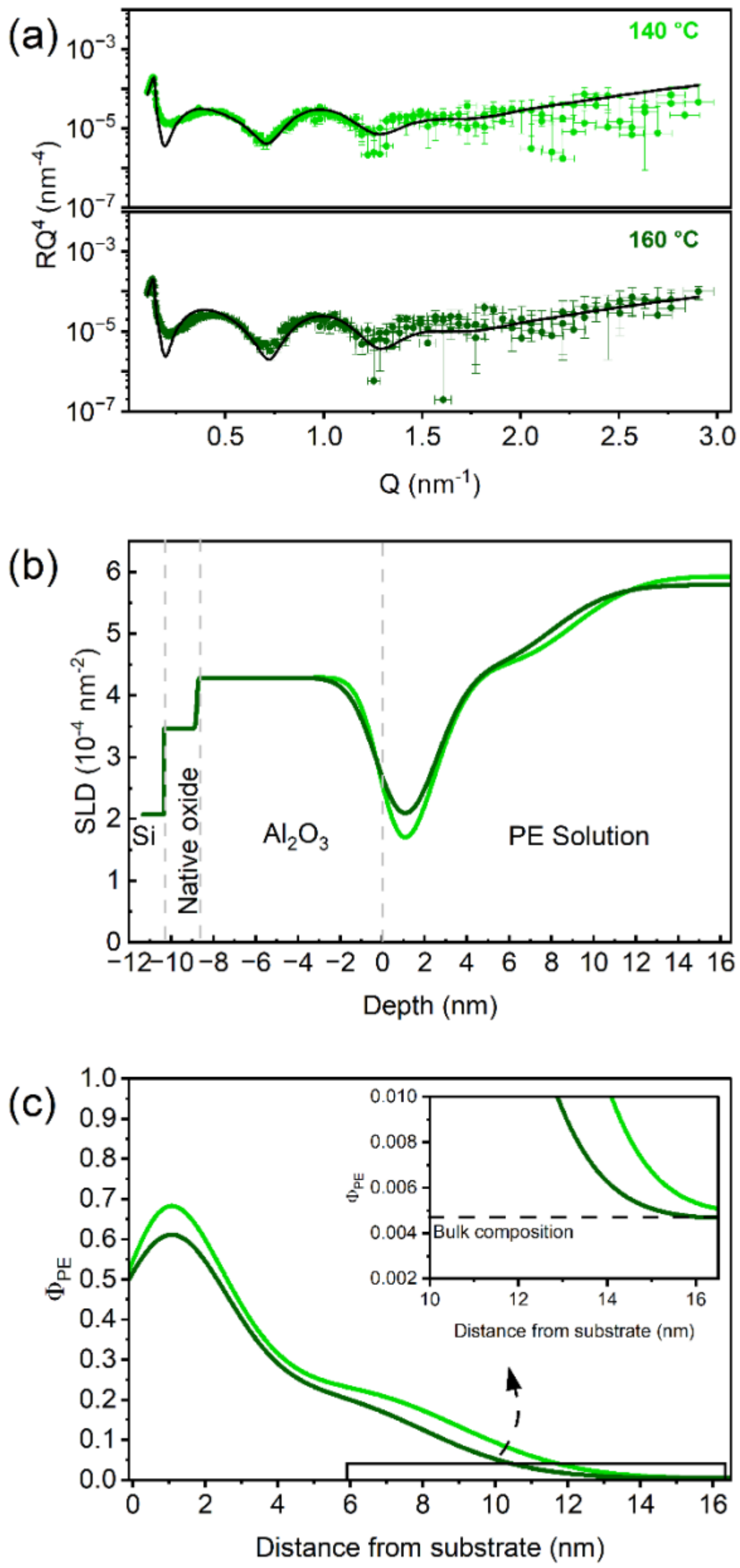

Figure S8. (a) Neutron reflectivity (NR) data (symbols) and model fits (solid lines) for 0.4 wt % 207 kg/mol PE in dC_{14} adsorbed on Al_2O_3 at 140 °C (bright green) and 160 °C (dark green). (b) SLD profiles normal to the interface obtained from the fitted models at 140 °C (green) and 160 °C (dark green). (c) Corresponding polyethylene volume fraction (Φ_{PE}) profiles as a function of distance from the Al_2O_3 surface. The inset highlights the decay of the polymer volume fraction toward the bulk solution.

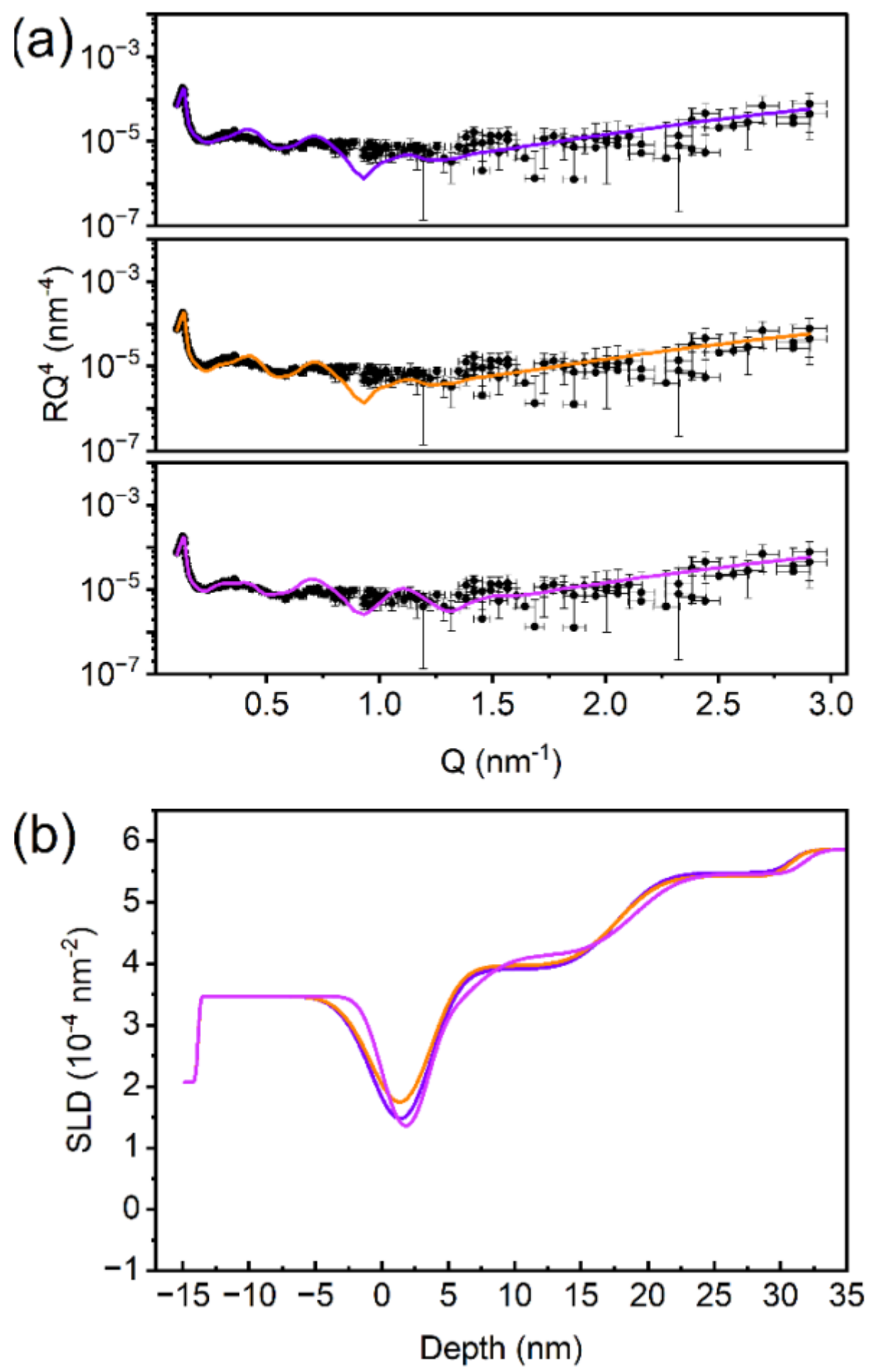

Figure S9. (a) Neutron reflectivity (NR) data (symbols) and alternative model fits (solid lines) for 0.4 wt % 34 kg/mol PE in dC_{12} adsorbed on SiO_2 at 140 °C (b) Corresponding SLD profiles normal to the interface obtained from fitting the data acquired at 140 °C. The fitting in general was robust irrespective of initial guesses for the number of layers, their SLD and thickness with the procedure outlined in the experimental section.
